# Hemolymph pressure-mediated friction in the tarsus of the desert locust (*Schistocerca gregaria*)

**DOI:** 10.1126/sciadv.aeg9427

**Published:** 2026-10-23

**Authors:** Jiahui Zhao, Zhendong Dai, Stanislav N. Gorb, Chuchu Li

**Affiliations:** ^1^ Jiangsu Provincial Key Laboratory of Bionic Functional Materials, College of Mechanical and Electrical Engineering, Nanjing University of Aeronautics and Astronautics, Nanjing, 210016, China.; ^2^ Functional Morphology and Biomechanics, Institute of Zoology, Kiel University, Kiel, 24098, Germany.

## Abstract

The desert locust (*Schistocerca gregaria*) performs high-acceleration jumps within milliseconds, relying not only on powerful muscles and spring-loaded hindlegs, but also on precise tarsi-substrate interactions. Although the mechanics of jumping have been extensively studied, the mechanisms enabling rapid attachment and detachment remain poorly understood. Here, we characterize the frictional properties of hindleg tarsi under controlled low-speed contact conditions targeting selected loading features associated with jumping. We hypothesize that locusts coordinate tarsal cuticular structures with internal hemolymph pressure to modulate contact mechanics. Using high-speed videography, a suite of advanced microscopic techniques, biomechanical experiments, and biomimetic prototypes, we provide evidence that hemolymph pressure modulates tarsal stiffness and acts together with structural asymmetry to produce direction-dependent frictional responses. This interplay may contribute to adaptive friction tuning associated with jumping. Our findings advance the understanding of locust locomotor biomechanics and suggest design principles for robotic systems requiring rapid and controllable surface engagement.

## INTRODUCTION

The desert locust (*Schistocerca gregaria*) can generate rapid jumps, reaching accelerations of over 200 m/s^2^ within tens of milliseconds (*1*, *2*). This remarkable performance depends not only on its powerful musculature and elastic energy storage mechanisms in the hindlegs (*1*, *3*–*8*), but also on the efficient leg-ground interaction during take-off. The distal part of the leg, the tarsus, plays a key role in mediating this interaction by generating friction for propulsion while allowing rapid detachment from the substrate (*2*, *9*–*11*).

Locusts typically jump at take-off angles below 45°, requiring substantial friction between the hindleg tarsi and the substrate to ensure horizontal propulsion. Two distinct structural components, rigid tibial spines and soft tarsal pads, mediate this interfacial interaction depending on substrate roughness (*2*, *12*–*15*). Extensive research has characterized the mechanical interlocking function of the spines on rough or compliant surfaces (*2*, *11*, *13*, *16*), but the contribution of the soft pads on smooth surfaces remains less clearly understood (*9*, *10*, *14*, *15*, *17*). Under smooth substrate conditions, the soft tarsal pads can establish proper contact with the substrate to generate sufficient friction for propulsion (*2*, *10*, *18*–*20*). During detachment, however, the same intimate contact may give rise to strong adhesion that can impede rapid release (*2*, *9*, *20*). Considering the extreme accelerations achieved during locust jumping, the tarsi must support both strong frictional engagement and rapid release. However, how the flexibly articulated tarsal pad system fulfills these opposing functional demands remains poorly understood.

To address this functional conflict, we investigated how locusts regulate contact mechanics under loading conditions associated with attachment and detachment on smooth surfaces. Given the presence of articulated tarsal segments equipped with soft, hemolymph-filled cuticular pads (*12*, *21*–*23*), we hypothesize that the locust employs internal hemolymph pressure to actively adjust tarsal stiffness and contact mechanics in response to functional demands. To test this hypothesis, we combined morphological characterization and high-speed observations of tarsal kinematics during jumping with controlled low-speed tribological experiments under defined pressure conditions. We characterized the frictional responses of the locust pads under both in situ and in vivo conditions and distinguished passive structural effects from active regulation. We further complemented these experiments with tarsal-inspired engineered prototypes of variable baseplate stiffness, designed to reproduce essential mechanical features of the biological system. By integrating biological and bioinspired engineering approaches, this study aims to elucidate mechanisms that may mitigate the apparent conflict between strong frictional engagement under compressive loading and rapid detachment, a challenge that remains insufficiently understood in friction-dominated attachment systems and bioinspired robotic interfaces (*24*–*30*).

## RESULTS

### Morphological and kinematic analysis of the desert locust hindleg

During high-speed motion after takeoff, desert locusts achieved stable adhesion to vertical smooth glass surfaces using their tarsi (movie S1.1). Together with the rapid detachment observed during take-off, this demonstrates that the hindleg tarsi can support contrasting functional demands associated with jumping and landing. To investigate the mechanisms enabling these functions, a detailed structural analysis of the tarsus was first performed. The microscopic images (Fig. 1 and fig. S1) revealed a segmented hinged structure of the desert locust hindleg tarsus. As shown in Fig. 1A, the proximal tarsomere (Tar1) features three distinct euplantular pads (Eu1–3), while subsequent segments (Tar2, Tar3) and pretarsus (Ptar) each possess a single pad: Eu4, Eu5, and an arolium (Ar), respectively. The unguitractor tendon (UT) is a critical component of the pretarsal mechanical system connecting claws with the muscle called retractor unguis (*31*). The CLSM image (Fig. 1B) further demonstrated the material heterogeneity of the tarsal cuticle. The red autofluorescence illustrated the highly sclerotized tips of claws and spines (Fig. 1B and fig. S1F). The blue autofluorescence in euplantulae (Eu1–5) and tarsal segments (Tar1–3) correlated with a large proportion of resilin or other elastic proteins (*32*–*34*), confirming the presence of compliant attachment structures. The intense blue autofluorescence at inter-segmental junctions indicated the structural flexibility of tarsal joints (fig. S1, D to F).

**Fig. 1.**
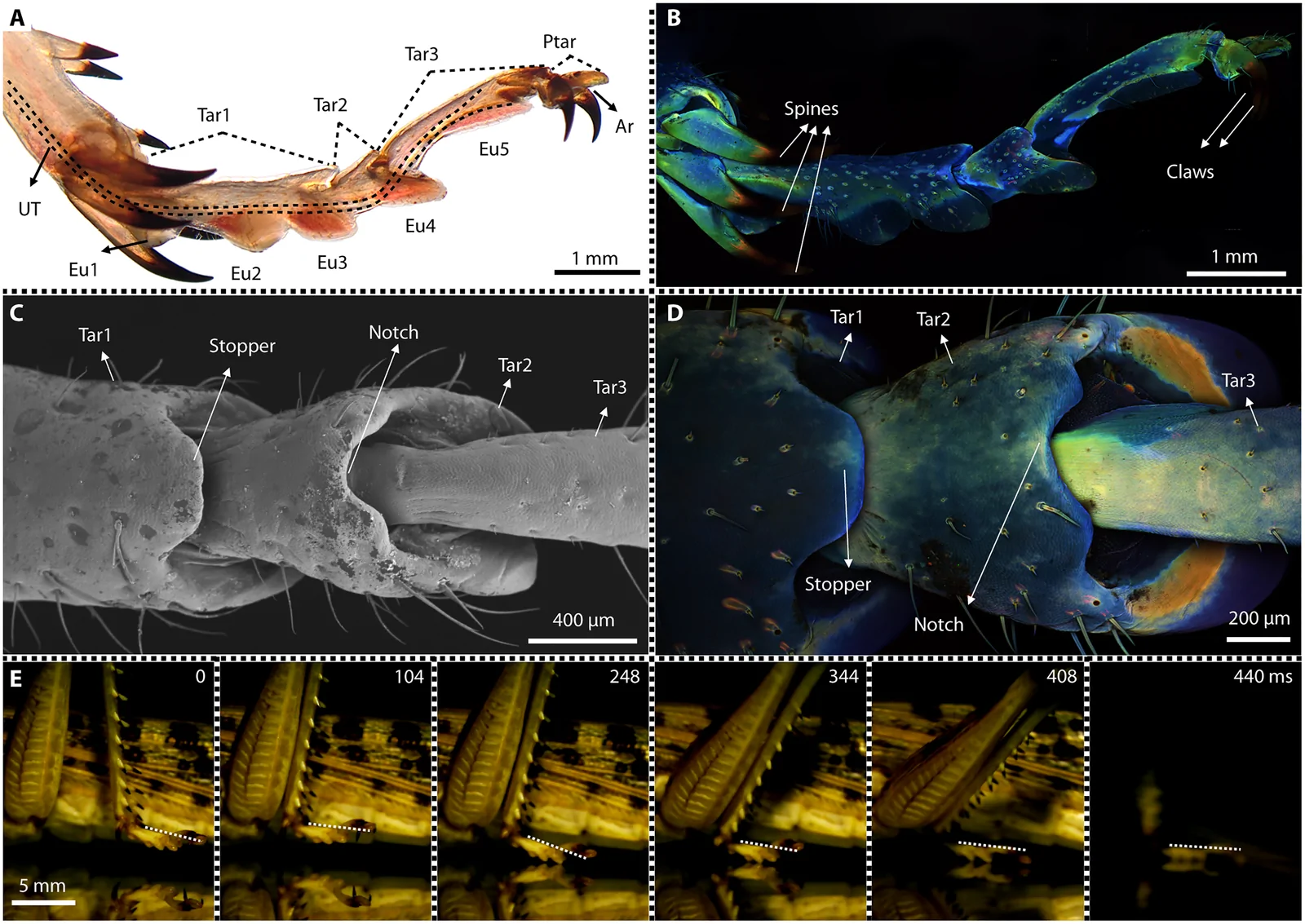
Hindleg tarsus morphology and jump kinematic modulation in the desert locust. (**A**) The tarsus consists of four articulated segments, each segment featuring at least one pad. (**B**) Confocal laser scanning microscopy (CLSM) reveals that the tarsus is a complex composite structure of highly sclerotized claws and non-sclerotized pads. (**C** and **D**) The dorsal cuticular morphology differs between adjacent tarsal segments, with the Tar1-Tar2 joint featuring a cuticular stopper and the Tar2-Tar3 joint containing a notch. (**E**) Biomechanical characterization of tarsal segment movement patterns: a white reference dashed line enables precise tracking of arolium movement.

The SEM (Fig. 1C) and CLSM images (Fig. 1D) revealed distinct articulation mechanisms across tarsal segments. A specialized cuticular stopper between the first (Tar1) and second (Tar2) tarsomeres suggests a biomechanical constraint limiting the dorsal flexion of Tar2. However, the dorsal cuticle between Tar2 and Tar3 features a significant intersegmental notch that facilitates a greater dorsal flexion of the Tar2-Tar3 joint. Furthermore, the distinct red autofluorescence observed at the distal area of Tar2 corresponds to a specialized sclerotized structure, which potentially acts as a stopper preventing ventral bending of Tar3 during the friction process. We employed high-speed videography to resolve the tarsal motions during locust jumping (Fig. 1E, movies S1.2 and S1.3). Notably, as evident in movie S1.2, the hindleg tarsal pads did not detach from the substrate simultaneously. Instead, the distal tarsomere lifted and detached first, causing the tarsal pads to separate sequentially from Eu4 to Eu1. This observation may have functional implications for rapid detachment. Previous studies have shown that adhesion in viscoelastic contacts can increase with retraction rate when the contact cannot relax or reduce its area sufficiently rapidly (*26*), and rate-dependent adhesion has also been reported for grasshopper tarsal pads (*17*). The sequential detachment observed here allows the contact area to decrease progressively rather than requiring multiple pads to separate simultaneously and may therefore reduce the instantaneous force required for detachment during rapid jumping.

To enable further analysis of the tarsal motions, it is essential to first understand how they are generated. The locust jumping apparatus comprises three functionally specialized segments: femur, tibia, and tarsus, forming an integrated kinematic chain supplemented with the muscle and tendon (Fig. 2A) (*21*). The tarsal motion was identified as consisting of four distinct motions driven by different systems (movies S1.3 to S1.6). These motions included flexion-extension cycles of the tibia (Fig. 2B) and tarsus (Fig. 2C), curling of the tarsus (Fig. 2D), as well as active lifting of the arolium (Fig. 2E). Motion I was driven by the dorsal femoro-tibial musculature (FTM1, levator of tibia) and the ventral femoro-tibial musculature (FTM2, depressor of tibia). Motion II was driven by the dorsal tibio-basitarsal musculature (TBM1, levator of tarsus) and the ventral tibio-basitarsal musculature (TBM2, depressor of tarsus). Motion III was driven by the femoro-pretarsal musculature (FPM) and the tibio-pretarsal musculature (TiPM, retractor of pretarsus) (*21*, *31*, *35*–*37*). Meanwhile, the tarsal joints were rich in elastic proteins (Fig. 1B), which endowed the tarsus with the ability to rapidly rebound after bending (movie S1.5). Motion IV, in contrast, represented a previously uncharacterized hydraulic control paradigm. Unlike the muscle-driven motions, this arolium-lifting is driven by an increase in the hemolymph pressure within the tarsal cavity (movie S1.6). Figure 1E shows a sequence of images capturing the jumping process, including both energy storage and release phases. The energy storage phase, hereafter called pre-jumping, starts from the onset of rapid tibial contraction (0 ms) to the initiation of jump propulsion (408 ms), while the energy release phase is jumping propulsion until take-off (440 ms). A white dashed line (Fig. 1E) positioned just above Tar1 serves as a reference to track the motion of the arolium. During the pre-jumping process, Tar1 and Tar2 function as an integrated unit, constrained by the cuticular stopper located at their junction. The time-series analysis revealed a distinct lifting of the arolium, which reached its maximal height at 248 ms and remained in this lifted position through subsequent frames until 344 ms. At 408 ms, however, the arolium abruptly dropped, reaching a position below its initial level at 0 ms. This sudden positional shift occurred just prior to take-off, coinciding with the onset of energy release. Hereafter, we refer to the elevated-arolium configuration as the “high-pressure tarsus” and the relaxed configuration as the “low-pressure tarsus,” to simplify subsequent descriptions.

**Fig. 2.**
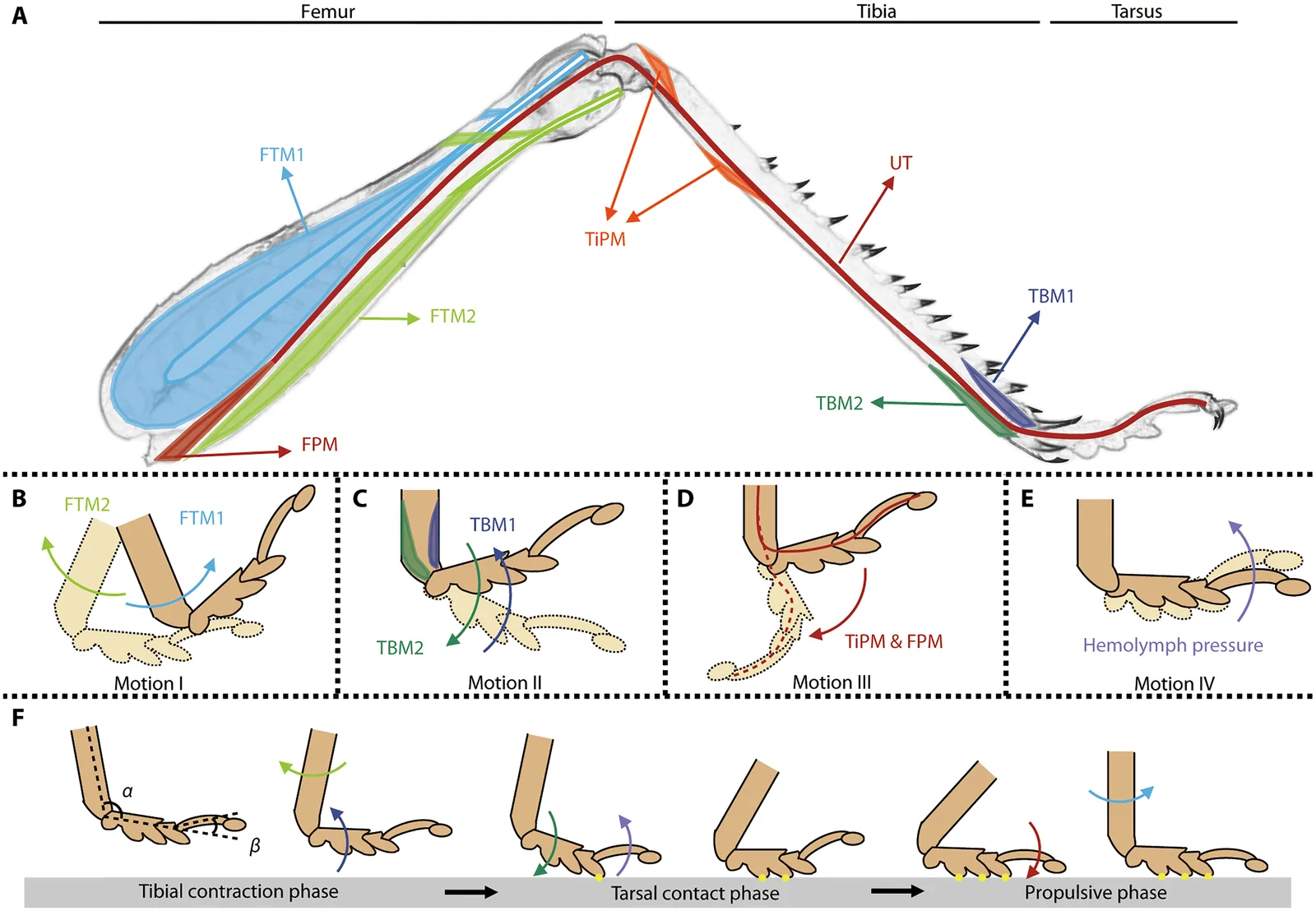
Schematic diagram of tarsal movement patterns and motion control system in the hindleg of desert locust. (**A**) Muscle anatomy of hindleg, including tarsus, tibia and femur (*21*). From *Principles of Insect Morphology*, by Robert E. Snodgrass, a Comstock Publishing Associates book published by Cornell University Press. Copyright 1993 by Cornell University. Used by permission of the publisher. (**B** to **E**) Tarsus involved motions and the corresponding control mechanisms: flexion-extension cycles of tibia (Motion I) and tarsus (Motion II), curling of tarsus (Motion III) and lifting of arolium (Motion IV). (**F**) Schematic representation of jump initiation, showing three sequential phases: tibial contraction, tarsal contact and propulsive phases.

Quantitative angular kinematics, based on the angle measurements of α (tibia-Tar1 angle) and β (Tar1/2-Tar3 angle, Fig. 2F), revealed the following sequential pre-jumping phases: (i) Tibial contraction phase: Clockwise tibial rotation reduces α from 109.7 ± 1.8° to 85.5 ± 2.3°, maintaining tarsal-substrate parallelism while increasing β from 15.2 ± 0.5° to 21.4 ± 1.1°. (ii) Tarsal contact phase: α and β concurrently increase to 98.3 ± 2.1° and 24.1 ± 1.5°, respectively, driving sequential pad attachment. Initial contact occurred via Eu4, while Eu5 and the arolium remained detached. Subsequently, progressive femoral depression shifts the load distribution (α to 70.3 ± 1.3°), transferring substrate contact from Eu4 to Eu3 and Eu2. (iii) Propulsive phase: Synchronized contact of Eu2–4 coincided with rapid arolium lowering. Tibial rotation (α to 95.3 ± 2.5°) generates peak acceleration, completing the jump within 38 ± 6 ms. Crucially, the β-angle increased during the tarsal contact phase, suggesting hydraulic involvement in tarsal configuration associated with jumping.

### Tribological characterization of in situ locust hindlegs

In a real jump, rapidly varying normal forces, shear loading, active muscular control, hemolymph pressure modulation, and sequential pad detachment are strongly coupled, making it difficult to disentangle cause-and-effect relationships. Therefore, we adopted a controlled sliding protocol with maintained contact to systematically quantify shear force, normal force, and real contact area, and investigated the regulatory role of hemolymph pressure in modulating the frictional behavior of the tarsus.

The stepwise in situ testing protocol was developed to investigate the pressure-dependent friction in the desert locust tarsus (Fig. 3A), with full experimental details provided in the Materials and Methods section. Figure 3B showed the recorded hemolymph pressure, contact area, normal force, and shear force against time for one test cycle. The orange curves and arrows showed the data for the low-pressure tarsus, while the red curves and arrows showed the data for the high-pressure tarsus. Based on loading conditions, the obtained data were grouped into sequential stages: preload (Step 1), friction right (Step 2), stop, friction left (Step 3), and stop. Here, Step 1 represented the application of normal load to the tarsus during the tarsal contact phase. Step 2 and Step 3 applied shear in directions corresponding to tarsal detachment during launch and tarsal attachment during jump preparation, respectively. Both frictions (left and right) were additionally split into two phases: (i) static friction phase (Stick), representing the stage where two contacting surfaces remain adhered to each other without slipping under the action of shear force, (ii) kinetic friction phase (Slip), representing the stage where the two surfaces in contact with each other have completely slipped. Motion cessation (Stop) was the stage at which the shear motion actuator stopped. It is important to clarify that the “normal force” in this study is not the normal force directly acting at the pad-substrate interface. In our setup, the normal force sensor is positioned above the tarsal-inspired structure and records the contact force between the dorsal side of the structure and the upper loading component.

**Fig. 3.**
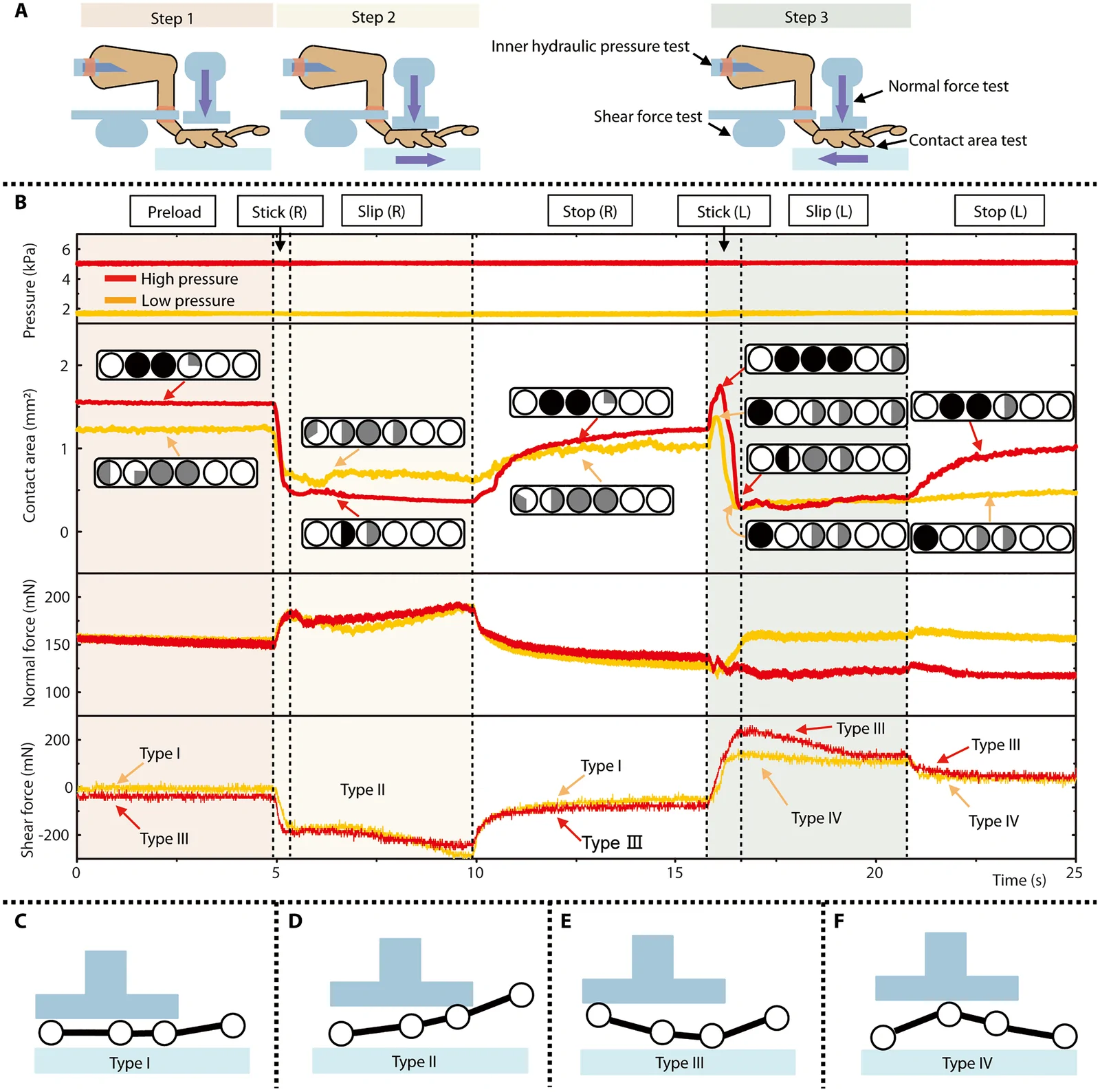
Tribological response of the in situ tarsus to the hemolymph pressure modulation. (**A**) Stepwise experimental design for tribological characterization of in situ locust hindlegs. (**B**) Combined recordings of pressure, contact area, normal force, and shear force during a complete pressure-modulated test cycle (150 mN preload) highlight dynamic changes in frictional behavior under low- and high-pressure conditions. The six circles in each rectangle represented the six tarsal pads (From left to right: Eu1–5 and Ar). The contact regions were indicated by geometric shapes with different gray levels, where a darker gray represented a higher contact pressure (see Fig. 6B for details). (**C** to **F**) 2D models of the tarsus illustrating four distinct contact configurations (Types I–IV) based on morphological and kinematic observations.

The pressure results showed that the hemolymph pressures remain constant for each testing stage (Fig. 3B), confirming that there was no leakage for both low- and high-pressure tarsi throughout the measurement. For the shear force, it should be noted that the sign convention only denoted the vector direction in this study, with negative values corresponding to rightward forces and positive values indicating leftward forces. While the actual contact area was dynamic, four types of configurations of the tarsus contacting were characterized as Types I–IV (Fig. 3, C to F and fig. S2, B to E), based on the experimental observations (movie S2). Type I showed contact primarily at Eu1–3/Eu1–4 with a limited contact area. Type II showed a distal tarsal lifting, while Type III showed an expanded contact region at Eu2–3/Eu2–4 (especially Eu2). Type IV showed lifting at the Tar1-Tar2 joint. These configuration variations arose from dynamic frictional loading. Types I and IV were generally observed in the low-pressure tarsus, whereas Type III occurred exclusively in the high-pressure tarsus. Type II appeared in both tarsi but only during Step 2 (Fig. 3, C to F and fig. S2, B to E).

The in situ testing results comparing high- and low-pressure tarsi mainly revealed the following key findings: (i) Increased hemolymph pressure enhanced the overall stiffness and the mechanical effects of structural asymmetry of the tarsus. (ii) During Step 3 or stationary contact with the substrate, increased hemolymph pressure improved tarsal contact. (iii) During Step 2, higher hemolymph pressure facilitated faster detachment, while lower hemolymph pressure maintained stronger contact with the substrate.

Finding (i) was primarily identified by comparing the frictional responses of low- and high-pressure tarsi in Step 1 and Step 3. During Step 1 (150 mN normal preload), a shear force of approximately 37 mN was detected in the high-pressure tarsus, whereas no measurable shear force was detected in the low-pressure tarsus. This difference did not result from the presence of morphological asymmetry itself, which occurred in both tarsi, but from the higher structural stiffness of the euplantulae under increased hemolymph pressure, which allowed the asymmetry to generate a measurable shear response. In addition, under a lower preload (10 mN; fig. S2A), this effect was not detectable in either tarsus. During Step 3, Stick (L), the contact area for both low- and high-pressure tarsi initially increased and then decreased with an increasing shear force. The initial increase was due to a progressive redistribution of interfacial stresses between the tarsus and substrate, wherein shear force caused a systematic shift in contact stress from proximal to distal tarsal segments. However, the subsequent decrease in contact area occurred through different mechanisms for low- and high-pressure tarsi. In the low-pressure tarsus, which had lower structural stiffness, the reduction in contact area resulted from the overall buckling of the tarsus. The buckling happened at the joint between Tar1 and Tar2, increasing the normal force and decreasing the attachment of Eu2–4 (Fig. 3F, fig. S2E, and movies S2.1 and S2.3). In contrast, the reduction in contact area for high-pressure tarsus resulted primarily from deformation of the pads (Eu2–4), leading to a decrease in normal force (Fig. 3E, fig. S2D, and movies S2.2 and S2.4). In brief, elevated hemolymph pressure increased overall tarsal stiffness, thereby suppressing joint flexion and enhancing structural asymmetry.

The contact area measurements from Step 1, Stop (R), Step 3, and Stop (L) further support Finding (ii). Under the same preload (150 mN, Step 1), the high-pressure tarsus exhibited a larger contact area (1.5 ± 0.03 mm^2^) compared to the low-pressure tarsus (1.2 ± 0.08 mm^2^). This difference can be attributed to the increased hemolymph pressure, enhancing the interfacial contact of Eu2–3. In addition, during Stop (R), the contact area of the high-pressure tarsus recovered more rapidly and completely than that of the low-pressure tarsus. It should be noted that although liquid lubrication promoted contact recovery, the area did not fully return to the Step 1 level due to the pad deformation. During Stop (L), only the high-pressure tarsus showed recovery, which is slightly different from Stop (R), where both low- and high-pressure tarsi partially recovered. The lack of recovery in the low-pressure tarsus resulted from global buckling deformation (Type IV), which diminished the effect of liquid lubrication. In Stick (L), the dynamic contact area of the high-pressure tarsus remained consistently larger than that of the low-pressure tarsus. Although both eventually decreased to similar levels, the different contact configurations led to a significantly higher shear force in the high-pressure tarsus (260.81 mN) compared to the low-pressure condition (161.52 mN). Furthermore, under a 10 mN preload (fig. S2A, Step 3), the high-pressure tarsus also showed a larger contact area and a higher maximum friction force (206.65 mN vs. 179.57 mN), confirming that elevated pressure improves tarsal contact.

In Step 2, the high-pressure tarsus demonstrated superior detachment performance, while the low-pressure tarsus tended to maintain contact [Finding (iii)]. At this stage, both low- and high-pressure tarsi exhibited a Type II configuration, in which leftward shear caused the distal tarsal segments (Ptar, Tar3, and Tar2) to lift upward, leading to progressive detachment from the substrate and a gradual decrease in contact area. However, the contact area of the high-pressure tarsus decreased more rapidly and extensively than that of the low-pressure tarsus, indicating that elevated hemolymph pressure facilitates easier detachment. During the Stick (R) of 10 mN preload (fig. S2A), the contact area of the low-pressure tarsus increased rather than decreased, whereas that of the high-pressure tarsus continued to decrease. Meanwhile, the maximum shear force for the high-pressure tarsus in Step 2 (118.28 mN) was significantly lower than that of the low-pressure tarsus (244.64 mN), indicating that low-pressure tarsi maintain stronger frictional coupling with the substrate under reduced preload conditions.

### Tribological characterization of in vivo locust hindlegs

To characterize the actively controlled friction in the hindleg tarsi of living locusts, the in vivo testing protocol (Fig. 4A) necessarily omitted direct hemolymph pressure measurements, which were implemented in the in situ testing protocol (Fig. 3A). The measured contact area, normal force, and shear force were analyzed following the previous methodology. As shown in Fig. 4B and fig. S3A, the dark-highlighted regions marked active modulations of the tarsal response to external stimulation, which were controlled by muscles and hemolymph pressure. Furthermore, although the tarsus of living locust still exhibited four distinct configurations (Fig. 4, C to F and fig. S3, B to E), the tarsal contact pattern of each configuration in in vivo testing would be slightly different from that in in situ testing due to active reactions of the living locust. For instance, the arolium may exhibit substrate contact under the influence of FPM and UT in the Type I configuration; elevating hemolymph pressure and contracting TBM2 elicited comparable frictional responses (Type III), particularly during Step 2, Stop (R), and Step 3.

**Fig. 4.**
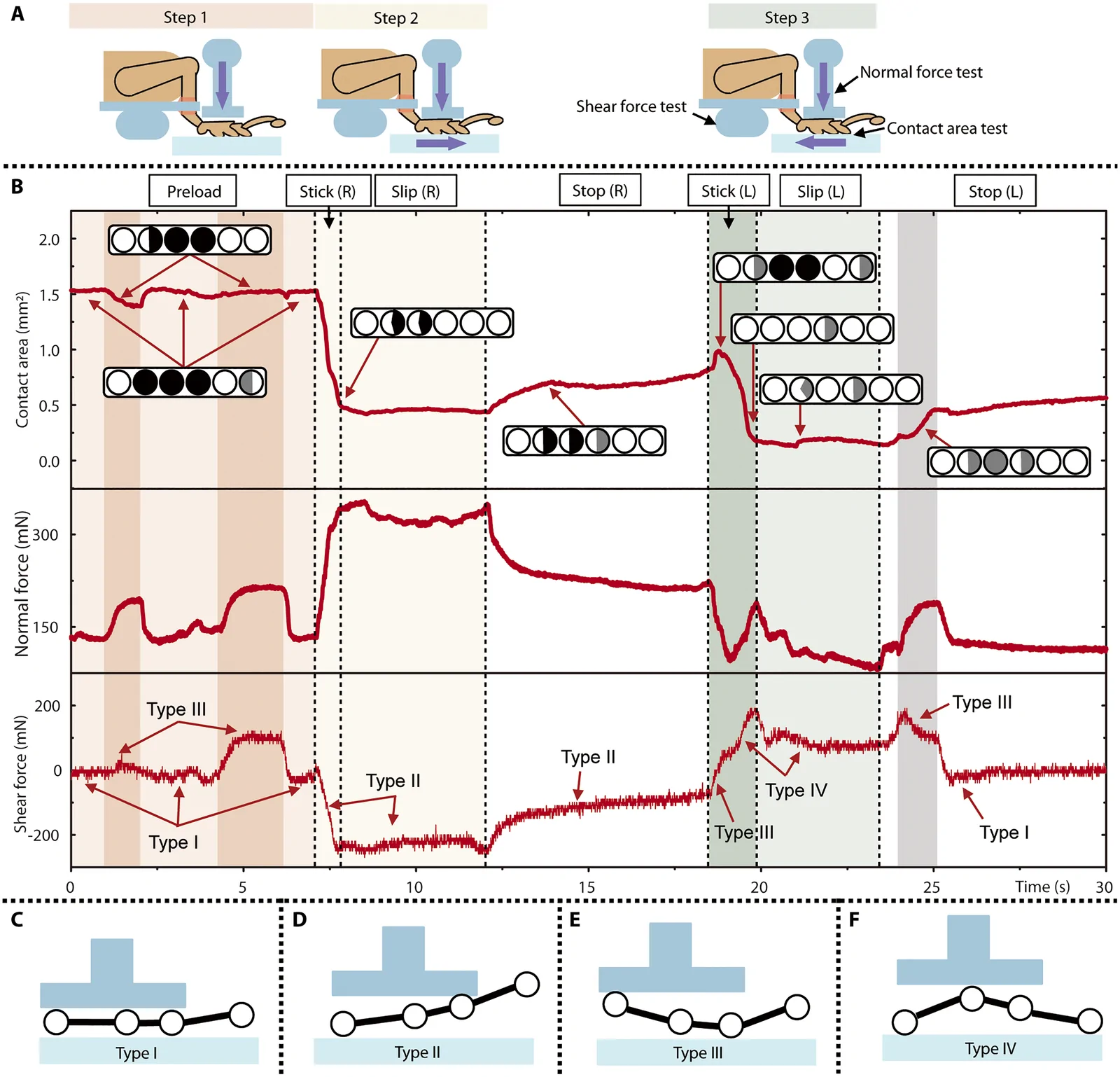
Tribological response of the in vivo tarsus to autologous hemolymph pressure modulation. (**A**) Stepwise experimental design for tribological characterization of the in vivo tarsus. (**B**) The impact of dynamic modulation of autologous hemolymph pressure on variations in tarsal contact area, normal forces, and shear forces throughout a single testing cycle (150 mN preload). (**C** to **F**) 2D model of tarsi with four distinct configurations as Types I–IV.

In the in vivo experiments, active control of the tarsus was clearly verified under four distinct conditions. First, when the tarsus was subjected to excessive normal constraints, the living locust actively increased tarsal hemolymph pressure to resist the load and prevent tissue damage. For example, applying a 150 mN preload during Step 1 (Fig. 4B) induced several active biomechanical responses in the tarsus (movie S3.1), whereas no such responses were observed under a 10 mN preload. The active hemolymph controls were confirmed by the arolium lifting movement, which then led to an increased normal force. Second, during Step 2, the living locust actively enhanced tarsal-substrate contact and resisted slipping. During the Slip (R) under a 10 mN preload (fig. S3A; movie S3.2), the in vivo tarsus exhibited two instances of active friction enhancement, accompanied by simultaneous increases in contact area, normal force, and shear force. According to mechanical analysis, this active control attempting to resist slippage may be achieved by contracting FTM1 and TBM2. For the Slip (R) under a 150 mN preload, the active control was not evident due to excessive normal load. Third, when the frictional motion stopped, the living locust actively controlled the recovery of contact between the tarsus and the substrate, as shown in the Stop (L) of Fig. 4B and Stop (R) of fig. S3A. Fourth, during Step 3 of both 150 mN and 10 mN preload, the variations in contact area indicated that the tarsal configuration transitioned from Type III to Type IV. The reason for the living locust initiating this active control might be that excessive friction loads could cause discomfort to the tarsus and pose a risk of instability. However, due to its complexity, the actively controlled friction at this stage will be discussed in detail in the following chapter.

In summary, it remained challenging to definitively attribute the active biomechanical responses observed in the in vivo tarsus to a single mechanism. Nonetheless, these observations demonstrate that living locusts actively modify tarsal contact mechanics in response to external loading.

### Tribological comparison between in situ tarsus and in vivo tarsus

To investigate the correlation between hemolymph pressure and the tribological characteristics of locust hindleg tarsi, we established functional relationships between normal and shear forces for both in situ tarsus (Fig. 5, A to D and fig. S4, A to D) and in vivo tarsus (Fig. 6, A to D and fig. S4, E to H) during the Step 2 [Stick (R) and Slip (R)] and Step 3 [Stick (L) and Slip (L)], respectively. Since the normal force varied in response to shear motion of the substrate, it served as an indicator of changes in tarsal configuration and apparent stiffness. Therefore, in Figs. 5, 6, and fig. S4, the shear force was defined as the independent variable and the normal force as the dependent variable. The data demonstrated a predominantly linear correlation between shear force and normal force, with the characteristic slopes of linear fits as shown in Figs. 5, 6, and fig. S4.

**Fig. 5.**
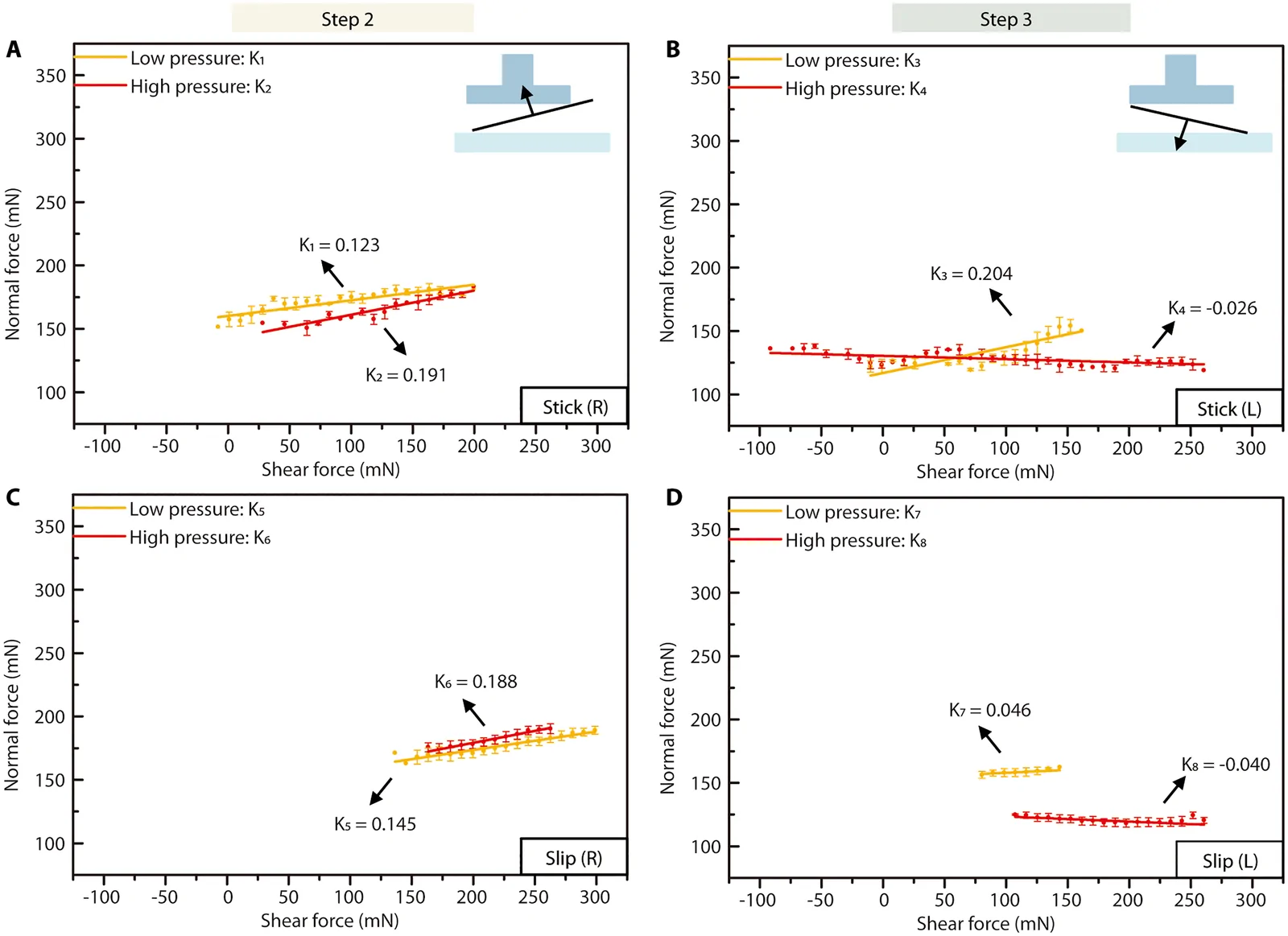
Normal-shear force relationships of in situ tarsi under 150 mN preload. The relationships between the normal forces and the shear forces of the in situ tarsus during (**A**) Stick (R), (**B**) Stick (L), (**C**) Slip (R), and (**D**) Slip (L).

**Fig. 6.**
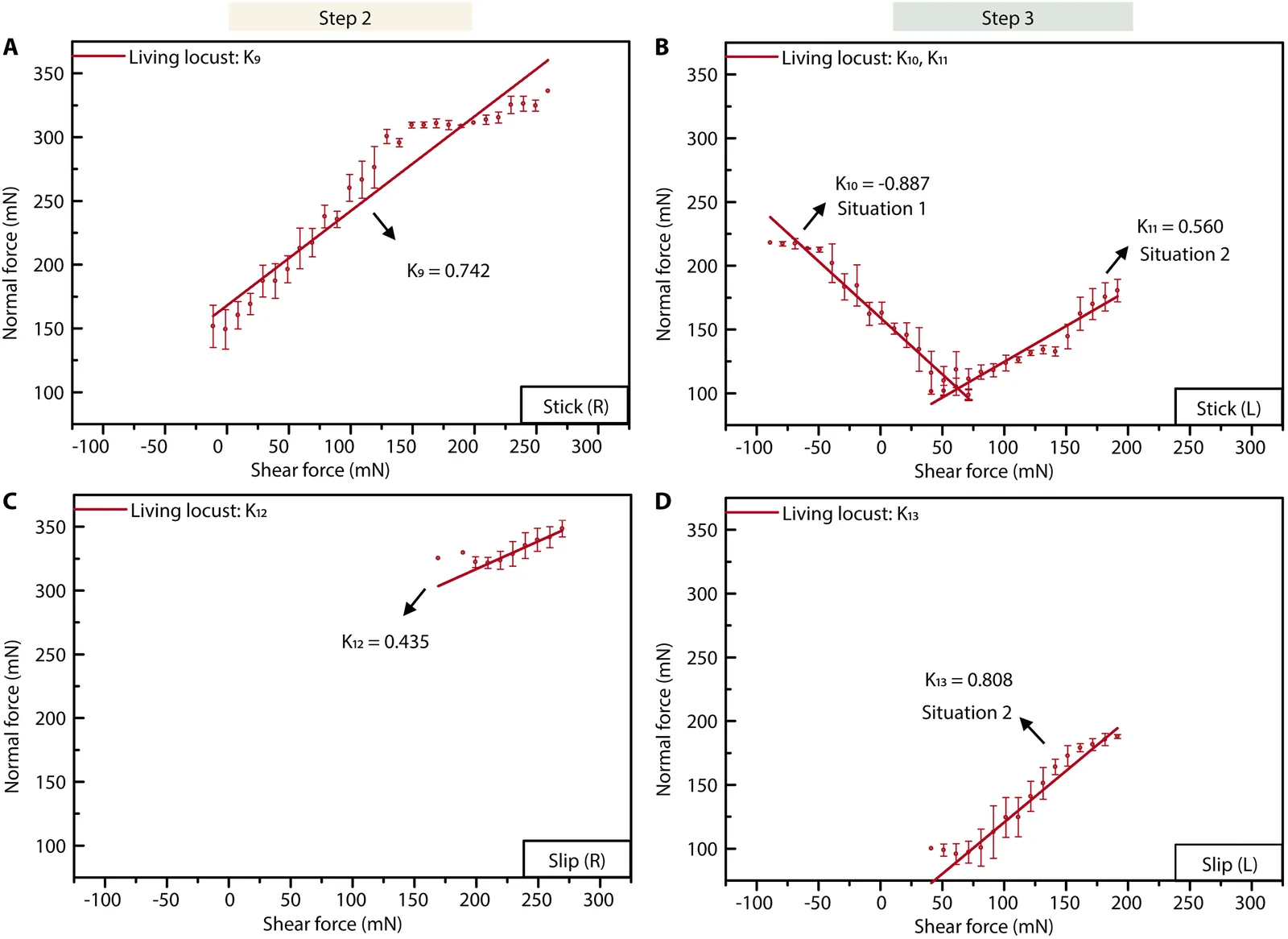
Normal-shear force relationships of in vivo tarsi under 150 mN preload. The relationships between the normal forces and the shear forces of the in vivo tarsus during (**A**) Stick (R), (**B**) Stick (L), (**C**) Slip (R), and (**D**) Slip (L).

In the in situ tests, across both preload and pressure conditions, all slopes in Step 2 showed positive values. When the preload was low (10 mN), tarsus with increased hemolymph pressure slipped more easily due to weaker adhesion (*10*). Consequently, the lifting effect of the distal tarsomere under rightward shear force was inhibited (normal force increases slowly), resulting in a smaller slope (fig. S4, A and C). When the preload was high (150 mN), the tarsus could make sufficient contact with the substrate, eliminating insufficient adhesion. In this case, the tarsus with increased hemolymph pressure exhibited higher stiffness, so the lifting effect of the distal tarsomere under rightward shear force was enhanced (normal force increases rapidly), yielding a larger slope (Fig. 5, A and C). In Step 3, the slopes of the low-pressure tarsus always showed positive values, while those of the high-pressure tarsus showed negative or nearly zero values. All these phenomena could be attributed to the enhanced overall stiffness of the hinged tarsal structure caused by increased hemolymph pressure, which restricts joint mobility and therefore leads to main effects. During Step 2, the higher stiffness enhances the tarsal leverage response and increases the peeling speed, resulting in a larger slope (Fig. 5A). During Step 3, it prevents tarsal buckling and redirects the resultant force of the tarsus toward the lower left, leading to a zero or negative slope (Fig. 5B).

During the in vivo testing, the slopes in Step 2 were larger than those in the in situ testing, which could be attributed to the FTM1 and TBM2 of living locusts facilitating friction during this stage. Interestingly, a transition of slopes occurred in Step 3 and showed clear differences between the two preloads (see Fig. 6B and fig. S4F, as well as Fig. 6D and fig. S4H). When applying a high preload (150 mN), the transition occurred at Stick (L) (Fig. 6B), showing a change from high-pressure tarsus (Situation 1) to low-pressure tarsus (Situation 2). However, when the preload was 10 mN, the slope experienced two transitions (figs. S4, F and H). The first transition occurred between Stick (L) and Slip (L), manifesting as the high-pressure tarsus change from Situation 1 to Situation 3. A probable reason for this transition is the contraction of TBM1, which leads to the lifting of the tarsus. The second transition occurred at Slip (L), where the slope changed from negative to positive, indicating the tarsus transitioned from a high-pressure state (Situation 3) to a low-pressure state (Situation 2).

These results further demonstrate the fourth condition of active control mentioned above, which the locusts modulate according to the degree of frictional loading. When the frictional load became excessive, the locust rapidly reduced hemolymph pressure to protect the attachment apparatus, whereas under insufficient frictional load, it enhanced tarsal attachment by contracting FTM1 and TBM2 or increasing hemolymph pressure.

### Tribological analysis of biomimetic tarsal-inspired structures

To verify the core hypothesis that structural stiffness governs tribological behavior in hinged attachment systems, we designed a series of simplified tarsal-inspired structures implementing controlled baseplate stiffness. The experimental protocol maintained methodological consistency with previous stepwise biomechanical testing (Fig. 7A). The contact area results of tarsal-inspired structures exhibited five distinct configurations as Types AI–AV (Fig. 7, C to G, fig. S5, B to F, and movie S4), where Types AI–AIV corresponded to Types I–IV of locust tarsus, respectively. The low-stiffness tarsal-inspired structure with negligible rotational stiffness at its joints exhibited antagonistic segment kinematics under applied loading, whose adjacent joints rotated in opposing directions (Type AV). The tribological characteristics of tarsal-inspired structures with different stiffness were compared (Figs. 7 and 8 and fig. S5).

**Fig. 7.**
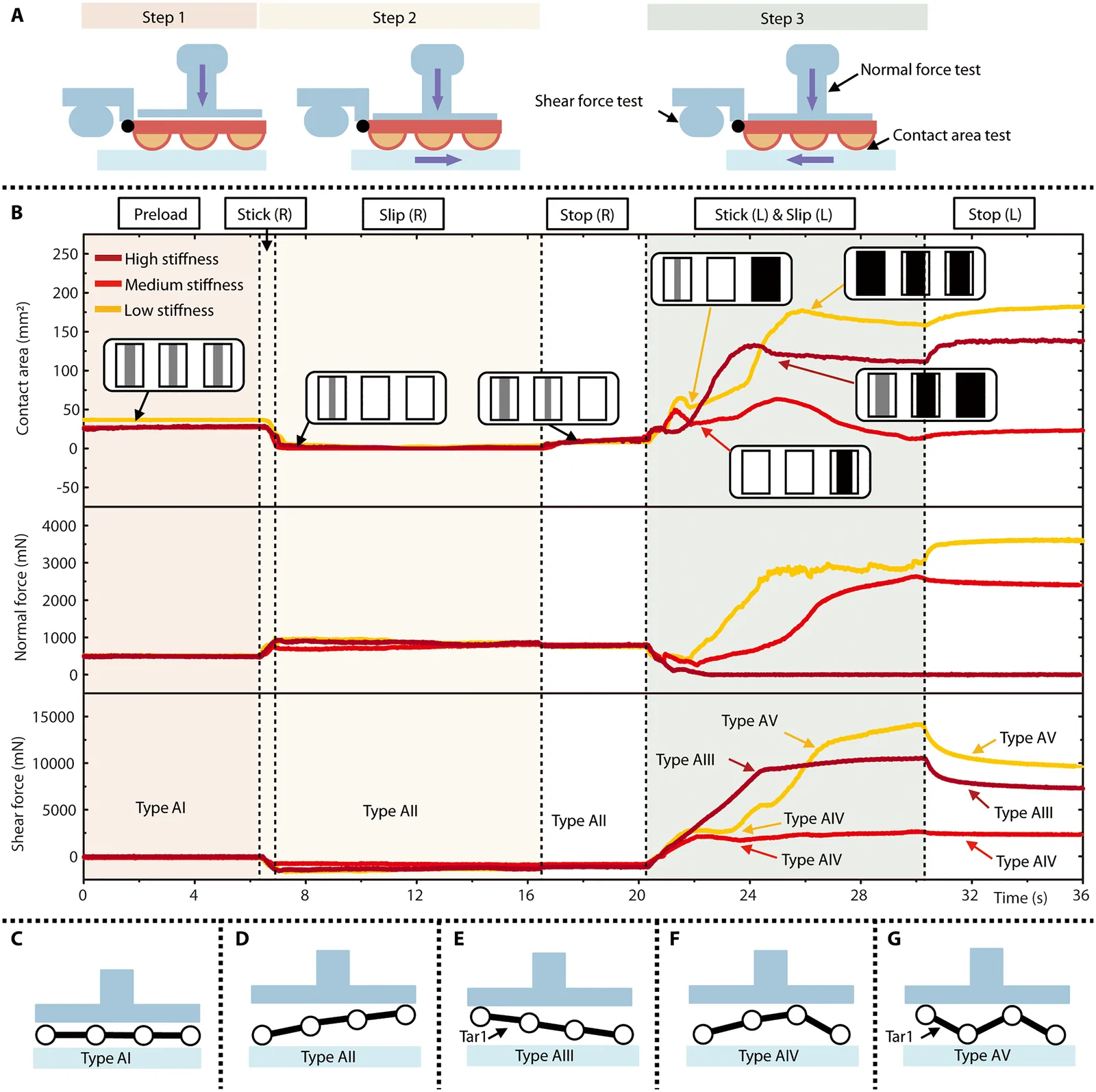
Tribological response of tarsal-inspired structures with variable stiffness of baseplate. (**A**) Stepwise experimental design for tribological characterization of tarsal-inspired structures. (**B**) Dynamic variations of contact area, normal force, and shear force to baseplate stiffness modulation in tarsal-inspired structures throughout a comprehensive experimental cycle (500 mN preload). The three rectangles in each larger rectangle represented the three tarsal-inspired pads (From left to right: Proximal pad, Middle pad, and Distal pad). The contact regions were indicated by geometric shapes with different gray levels, where a darker gray represented a higher contact pressure (see Fig. 6C for details). (**C** to **G**) 2D modeling of tarsal-inspired structures with five distinct configurations as Types AI–AV.

**Fig. 8.**
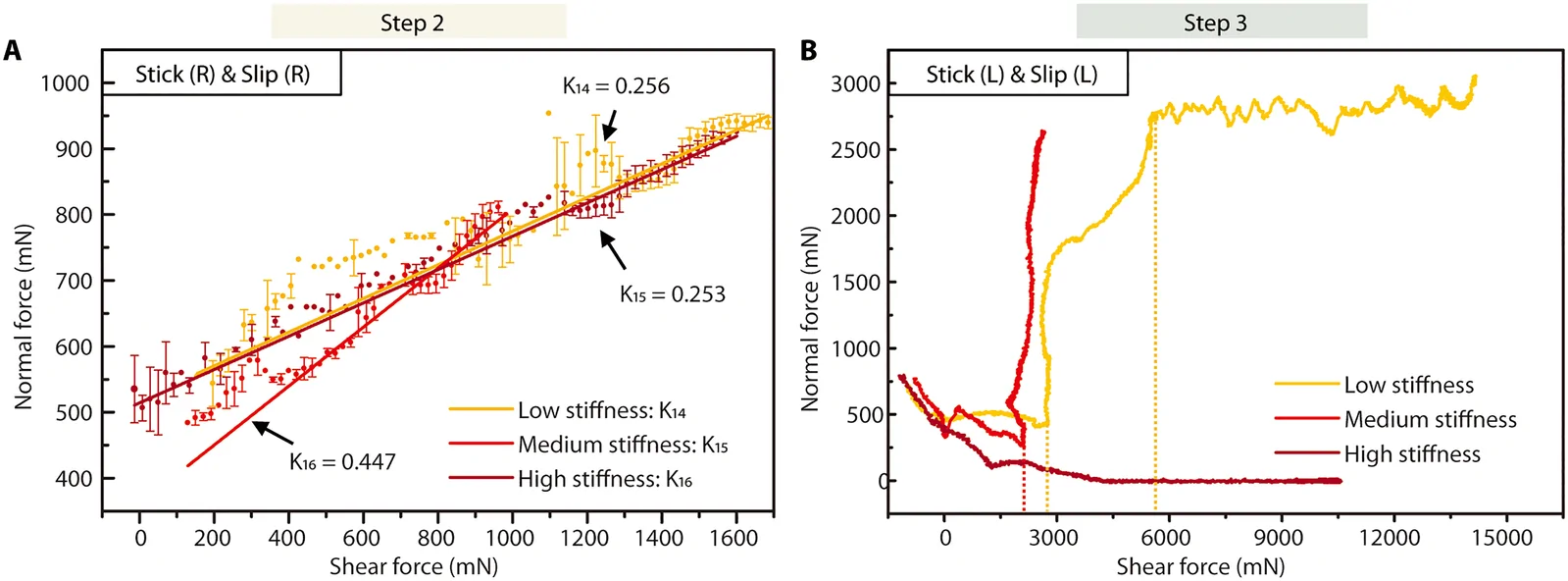
Normal-shear force relationships of tarsal-inspired structures under 500 mN preload. The effects of baseplate stiffness variations on normal-shear force relationships during (**A**) Step 2 and (**B**) Step 3.

As shown in Fig. 7B and fig. S5A, the tarsal-inspired structures exhibited distinct anisotropic friction behavior. During Step 1, Step 2, and Stop (R), all tarsal-inspired structures exhibited similar configurations (Types AI and AII), along with highly consistent contact area, normal force, and shear force, indicating that frictional behaviors in these stages were independent of structural stiffness. By comparison, the friction behaviors of tarsal-inspired structures exhibited significant differentiation in Step 3, resulting in the shear forces (particularly low- and high-stiffness structures) two orders of magnitude higher than those in Step 2. Notably, the drop of the measured normal force to zero in the high-stiffness structure during Step 3 does not indicate that the pads detach from the substrate. Instead, it indicates that the dorsal side of the structure loses contact with the upper loading component. Under leftward shear loading in Step 3, the high-stiffness structure is driven downward toward the substrate, producing stronger pad-substrate engagement. Once the structure is pressed against the substrate by the shear-induced moment, the upper loading component no longer bears compressive force, and the measured normal force decreases to zero. Thus, the zero normal force reflects a change in load transfer within the testing configuration, rather than loss of pad-substrate contact. As demonstrated by the subsequent analysis, this exceptional mechanical performance stemmed from increased structural stiffness.

During Step 3, we initially hypothesized that the high-stiffness structure would exhibit the highest frictional performance and the low-stiffness structure the lowest. However, the results did not support this expectation. As shown in Fig. 8B and fig. S5H, all tarsal-inspired structures exhibited negative slopes during the initial phase of Step 3, corresponding to the attachment of the pads to the substrate. Subsequently, the frictional behaviors of the high- and medium-stiffness structures followed the expected trends (Fig. 7B, fig. S5A, and movie S4), namely, the high-stiffness structure maintained configuration Type AIII, whereas the medium-stiffness structure transitioned to Type AIV due to buckling. In contrast, the low-stiffness structure, although expected to undergo even greater buckling, transitioned from Type AIV to Type AV due to opposite loading between adjacent joints, thereby shifting the optimal contact toward the proximal pad (Fig. 7G) and enhancing frictional performance. The opposite loading behavior in the low-stiffness structure was also evident under low-preload conditions (50 mN, fig. S5F), suggesting that this effect is an intrinsic feature of the compliant design rather than a response to excessive normal loading.

## DISCUSSION

Our findings indicate that hemolymph pressure contributes to the adaptive frictional performance of the tarsus by acting in concert with a structurally asymmetrical hinge system. High-speed videography revealed elevation of the arolium in association with the transition to jumping, while pressure-manipulation experiments showed that this elevation is induced by increased hemolymph pressure. These observations suggest that hemolymph pressure is elevated during the transition from a non-jumping to a jumping state. Under the tested conditions, this elevated pressure supported both contact formation and detachment. Hemolymph pressurization further enhances the mechanical effects of the tarsus morphological asymmetry by modulating joint stiffness and pad deformation during contact. Structurally, this asymmetry originates from three complementary features. First, the tibio-tarsal articulation in locusts forms an asymmetric L-shaped geometry (Fig. 1A), in contrast to the T-shaped designs often adopted in robotic jumping mechanisms (*2*, *30*, *38*, *39*). While the T-shaped configuration enhances static stability, the L-shaped morphology optimizes torque transfer during pad engagement and facilitates rapid tarsal detachment during take-off. Second, the tarsal cuticle displays a pronounced dorsal-ventral material gradient, combining a stiff dorsal exoskeleton with compliant ventral euplantulae (*9*, *10*). Such a gradient allows the ventral pads to deform and establish effective contact with the substrate under normal load, while the stiff dorsal side maintains sufficient structural rigidity to prevent system failure. This mechanical stability is further reinforced by localized sclerotization near Eu4 (Fig. 1D). Similar material heterogeneity has been widely recognized as a functional adaptation in other parts of the locust leg, where graded stiffness enables local deformation without compromising overall structural rigidity (*6*–*8*).

Third, most insects with flexible pads possess only one euplantula per tarsal segment, such as cockroaches (*40*), stick insects (*35*) and mantises (*20*). However, locusts have three euplantulae on the first tarsal segment and one on each of the other tarsal segments. An uneven distribution of euplantulae has also been observed in other grasshoppers, for example, bush crickets that have two euplantulae on the first tarsal segment (*41*). Biomimetic tribological analyses of tarsal-inspired structures confirmed that this hinge configuration combines the advantages of both high- and low-stiffness designs. Optimal frictional performance occurred when Tar1 tilted downward toward the substrate, allowing its pad to establish efficient contact, while Tar2 primarily acted as a supporting link transmitting shear force (Fig. 7, E and G and fig. S5, D and F). The greater number of pads on Tar1 further amplifies this effect, suggesting that the uneven distribution of euplantulae is functionally advantageous for frictional engagement. Furthermore, the downward tilting of Tar1 in the real locust system is actively assisted by the TMB2 muscle, whereas the other tarsal joints lack direct muscular control. This indicates that local muscle activity works in concert with hemolymph pressurization to fine-tune frictional engagement.

Our results demonstrate that hemolymph pressure modulates pad contact and joint stiffness, thereby producing direction-dependent frictional responses through the structurally asymmetric tarsus. In the in situ tests, the low-pressure tarsi exhibited buckling and low shear forces (∼162 mN) during attachment, whereas high-pressure tarsi preserved pad contact and reached significantly higher friction (∼261 mN). Elevated hemolymph pressure also facilitated faster detachment from the substrate after force application, indicating that the hydraulic system enhances not only attachment but also detachment. This hydraulic modulation represents an additional level of active control beyond muscular actuation. Classical studies have established the muscular basis of tarsal motion (*3**,* *21**,* *35**–**37**,* *42*), yet insects and other arthropods are also known to rely on hydraulic mechanisms for actuation, such as pad deployment in ants (*23*), leg extension in spiders (*43*), and antennal adjustments in insects (*44*). Our findings extend this concept to tarsal friction control, showing that hemolymph pressure functions as a dynamic actuator that cooperates with the muscular system to meet different frictional demands.

Broader theoretical perspectives on animal hydraulics (*45*, *46*) suggest that pressure-muscle coupling represents a general biomechanical strategy. In locusts, this coupling provides a rapid and reversible means of adjusting frictional engagement, explaining why tarsi lacking hemolymph support, as noted by Gorb *et al.* (*31*), recover more slowly and incompletely from flexion. Our results thus identify hemolymph pressurization as the key integrative mechanism linking tarsal structure, muscle action, and frictional performance. Beyond friction, the hydraulic modulation of stiffness also acts as a load-responsive protection mechanism. In vivo tests confirmed that the living tarsus can actively switch within milliseconds from a high-pressure, stiff configuration to a low-pressure, compliant one when the shear forces become excessive, for example, during the Stick (L) phase (Fig. 4B). This dual functionality prevents mechanical overload of the euplantulae and ensures efficient energy transfer during the explosive thrust of jumping.

Our tarsal-inspired structure experiments reproduced key tribological features of the locust tarsus by systematically varying structural stiffness. Here, the experimental design deliberately reflects the L-shaped limb geometry of the locust, allowing us to examine how structural asymmetry influences frictional engagement and detachment under a fixed-pivot boundary condition. As a result, shear forces naturally generate asymmetric moments that can either promote lifting (detachment) or enhance contact (engagement), depending on the loading direction. The high-stiffness structure replicated the frictional behavior of high-pressure tarsi, enhancing contact and frictional engagement under the attachment-related loading direction while facilitating lifting and detachment under the opposite direction. This interpretation is further supported by observations of the biological system. High-speed recordings (movies S1.2-S1.3) show that shear loading can induce lifting and sequential detachment of distal tarsal segments, while in other phases it promotes enhanced contact and frictional engagement. Thus, the directional response observed in the high-stiffness structure is consistent with the functional behavior of the locust tarsus. However, the fixed-pivot boundary condition in this study differs from that of a freely moving locust. During natural jumping, translation and rotation of the proximal leg and body may partly relieve the wedging effect generated under the substrate engagement. Therefore, rigid fixation may amplify the directional asymmetry measured in our experiments, and the magnitude of this effect may differ from that occurring during natural jumping.

The medium-stiffness structure most closely approximated the low-pressure tarsi. However, its performance was limited because only the distal pad maintained substrate contact while the middle pad lifted off, resulting in the smallest contact area and the lowest shear force among the tested tarsal-inspired structures. These results indicate that frictional performance is highly sensitive to how loads are distributed across pads rather than to joint stiffness alone. When the distal end of the medium-stiffness baseplate deflects under localized stress, its semi-flexible joints with non-zero stiffness fail to provide sufficient support for stress transmission, which in turn forces the middle and proximal pads to lift due to insufficient compliance. In contrast, the low-stiffness structure exhibited the highest friction among all tested structures. This enhancement resulted from joint collapse, which maximized pad-substrate contact area, and was further amplified by a progressive stiffening during later stages of shear (Type AV). This behavior enabled strong propulsion friction even without preloading forces, achieving performance even exceeding that of high-stiffness structures. However, this situation is unlikely to occur in natural insect tarsi, because real joints exhibit non-negligible motion resistance (*9*, *12*, *31*). Instead, biological systems are constrained by evolutionary trade-offs, where joint stiffness and resistance are optimized to balance mobility and stability rather than maximizing friction (*43*).

Additionally, several limitations of the present study should be acknowledged. First, all mechanical tests were conducted under displacement-controlled loading, which fundamentally differs from the force-regulated locomotor mechanics of real locust jumping, where dynamic contact forces are actively modulated by muscular and hydraulic regulation. Second, contact area measurements were acquired under controlled low-speed conditions, which cannot fully replicate the rapid interfacial deformation and contact variation that occur during real high-acceleration jumping motions. Third, the biomimetic structures implemented here reproduce only the essential hinge mechanics of the tarsus but do not incorporate the full material complexity of biological pads. Addressing these limitations will require future studies integrating real-time pressure measurements during natural jumping as well as more advanced bioinspired materials.

From an engineering perspective, this divergence offers a useful design framework. Next-generation jumping robots may benefit from artificial hinges that can deliberately exploit such low-stiffness states to maximize propulsion forces. Future advances in adaptive materials, such as stiffness-tunable polymers (*47*), stiffness-gradient composites (*48*), or shape-memory polymers (*49*), may eventually enable artificial actuators to replicate the stiffness modulation capabilities of hemolymph-pressurized insect tarsus while maintaining structural integrity.

## MATERIALS AND METHODS

### Animals

Adult desert locusts *Schistocerca gregaria* were obtained from commercial suppliers (ZOO & Co., Kiel, Germany), and maintained under controlled lab conditions (temperature: 27 ± 2°C; humidity: 30–40%; 12 hour: 12 hour light: dark cycles). As this study involved only the invertebrate species *Schistocerca gregaria*, approval by an ethics committee was not required under current EU and German regulations. Specimens underwent a 10-day quarantine/acclimatization phase prior to the experiments, during which they were monitored daily for behavioral indicators to confirm the absence of stress. Round lettuce was the main source of food provided to the locusts. Adult gregarious desert locusts exhibit significant sexual dimorphism in body size (*2*). To eliminate the potential confounding effects of body size on experimental results, only adults of a single sex were used in this study. Moreover, female desert locusts carry males on their backs during locomotion throughout the reproductive period (*50*). During this stage, females are unable to fly and rely solely on jumping for movement, which subjects the tarsi of their hindlegs to particularly demanding frictional interactions. Therefore, to better investigate how the condition of the hindleg tarsi influences friction performance, adult female desert locusts were chosen as the experimental model in this study.

### Visualization

Active adult locusts were euthanized using CO_2_, and then the hindlegs were cut off with a scalpel. The fresh tarsi of the hindlegs were subjected to standardized processing, which involved rinsing with 75% ethanol followed by mounting in glycerol (≥99.5%, Carl Roth GmbH & Co. KG, Karlsruhe, Germany) to achieve refractive index matching and minimize optical artifacts. The morphology of the hindleg tarsi was visualized through the stereomicroscope (SZX10, Olympus Corporation, Tokyo, Japan).

For further observation, the tarsus was air-dried and mounted on aluminum stubs with carbon leit tabs (G3347, Plano GmbH, Wetzlar, Germany), sputter-coated with gold-palladium (6–10 nm thickness) using a Leica EM sputter coater (SCD 500 High-Vacuum, Leica Microsystems GmbH, Wetzlar, Germany), and examined in a scanning electron microscope (SEM, TM3000, Hitachi High-Tech., Tokyo, Japan).

A confocal laser scanning microscope (CLSM, Zeiss LSM 700, Carl Zeiss Microscopy, Jena, Germany) was employed to characterize the material composition of the tarsal cuticle. The CLSM was equipped with four lasers (laser lines: 405, 488, 555, 639 nm) to excite the samples subsequently. Four emission filters transmitting 420–480 nm, ≥490 nm, ≥560 nm, and ≥640 nm were used to visualize the autofluorescence of the tarsal cuticle. The obtained CLSM images enable assessing the cuticular sclerotization level. Autofluorescence spectral profiles exhibited a correlation with sclerotization of cuticle: non-sclerotized regions showed dominant emission in the blue spectral region, intermediately sclerotized regions exhibited green-to-yellow fluorescence, while highly sclerotized cuticle showed intense red autofluorescence, corresponding to progressive crosslinking of cuticular proteins and phenolic tanning (*32*–*34*).

Videos of desert locusts stably adhering to vertical smooth glass surfaces after jumping were obtained using a high-speed camera (500 fps, RX10 III, Sony Group Corporation, Tokyo, Japan). The complete kinematic sequence of tarsal-substrate interaction during the pre-jumping and propulsion phases in the desert locust was captured using a high-speed camera (125 fps, Fastcam SA1, Photron, San Diego, California, USA) coupled with a cold light projector (Storz techno light 270, Karl Storz SE & Co. KG, Tuttlingen, Germany).

### Tarsal-inspired structures

In this study, the customized components were systematically categorized into rigid and flexible types based on their mechanical properties. The rigid components, which demonstrated superior mechanical strength, were fabricated using fused deposition modeling technology (3D printing, Original Prusa i3 MK3S Plus, 0.25 nozzle, Prusa Research a.s., Prague, Czech Republic) with conventional polylactic acid (Prusament PLA, Prusa Research a.s., Prague, Czech Republic). The flexible components were fabricated through a multi-stage manufacturing process combining molding techniques with thermal cutting (Thermocut 230/E, Proxxon GmbH, Föhren, Germany), employing liquid silicone rubber (Dragon Skin 20, Smooth-On Inc., Macungie, USA) and commercially sourced sponges.

As shown in fig. S6A, three tarsal-inspired structures with different stiffness were engineered based on the hinge principle of locust hindleg tarsus, specifically categorized as high-stiffness, medium-stiffness, and low-stiffness types to achieve differentiated mechanical performance. All tarsal-inspired structures featured three hemi-cylindrical frictional units, each comprising a uniform viscoelastic core (commercial sponge, radius: 5.0 ± 0.1 mm, width: 15.0 ± 0.2 mm) encapsulated by a smooth elastomeric membrane (Dragon Skin 20, thickness: 0.8 ± 0.1 mm, Smooth-On Inc., Macungie, USA). These frictional units were arranged in equidistant parallel alignment relative to their mounting baseplate, maintaining uniform spacing across all specimens. The whole baseplates of three tarsal-inspired structures have similar sizes (length: 48.0 ± 0.8 mm, width: 15.0 ± 0.2 mm, thickness: 4.0 ± 0.2 mm). The high-stiffness baseplate was fabricated through 3D printing using rigid polymers (Prusament PLA, Prusa Research a.s., Prague, Czech Republic), demonstrating negligible compliance (<0.5% strain) under the quasi-static loading regime employed in this study. The medium-stiffness baseplate consisted of a silicone rubber body (thickness: 3 mm ± 0.3 mm) composited with three thin 3D-printed tops (length: 15.0 ± 0.2 mm, width: 13.0 ± 0.2 mm, thickness: 1.0 ± 0.1 mm), which ensured that all baseplates had similar contact surfaces with the normal force sensor. The low-stiffness baseplate (length: 16.0 ± 0.5 mm, width: 15.0 ± 0.2 mm, thickness: 4.0 ± 0.2 mm) was fabricated through identical 3D printing parameters and assembled via screw-and-nut systems, featuring near-zero rotational stiffness at inter-segmental joints across the operational range. Figure S6, B and C presented the three-point bending test results of high-stiffness and medium-stiffness structures measured using a universal testing machine (zwickiLine, ZwickRoell GmbH & Co. KG, Ulm, Germany) with a support span of 40.0 mm. The central load of the high-stiffness structure reached 37.99 N at a deflection of 1.78 mm, whereas that of the medium-stiffness structure was only 0.32 N at a deflection of 1.94 mm.

### Experimental platform for friction characterization

Although direct measurement during natural jumping of locust would be ideal, this process is highly transient and strongly coupled. This makes it extremely challenging to simultaneously resolve force transmission, contact evolution, and internal actuation with sufficient spatial and temporal resolution. In particular, key measurement modalities involve inherent trade-offs. For example, frustrated total internal reflection (FTIR) imaging enables high-resolution visualization of the real contact area but requires weak illumination and is not compatible with high-speed recording (requires strong illumination) under dynamic conditions. Conversely, high-speed imaging captures rapid kinematics, but does not provide direct access to interfacial contact mechanics. As a result, it is not currently feasible to obtain all relevant variables within a single experimental framework during natural jumping. Instead of attempting to directly reproduce the full jumping dynamics, we deliberately adopt a mechanism-oriented strategy. Through controlled in situ experiments, in vivo observations, and simplified mechanical abstractions, we systematically examine how structural design and hemolymph pressure govern frictional engagement and detachment.

To systematically characterize the dynamic friction mechanisms of desert locust hindleg tarsus and tarsal-inspired structures, we developed a multifunctional tribological testing platform capable of synchronously measuring shear force, normal force, hemolymph pressure, and real contact area via FTIR (Fig. 9A and fig. S7). In biomechanical experiments, comparative friction analyses were conducted under two physiological conditions (Fig. 9A). In situ testing: Freshly dissected hindlegs with controlled hemolymph pressure modulation. In vivo testing: Restrained live specimens with natural pressure variations during friction. Additionally, the frictional properties of the three individual tarsal-inspired structures were tested and compared with the results of biomechanical experiments.

**Fig. 9.**
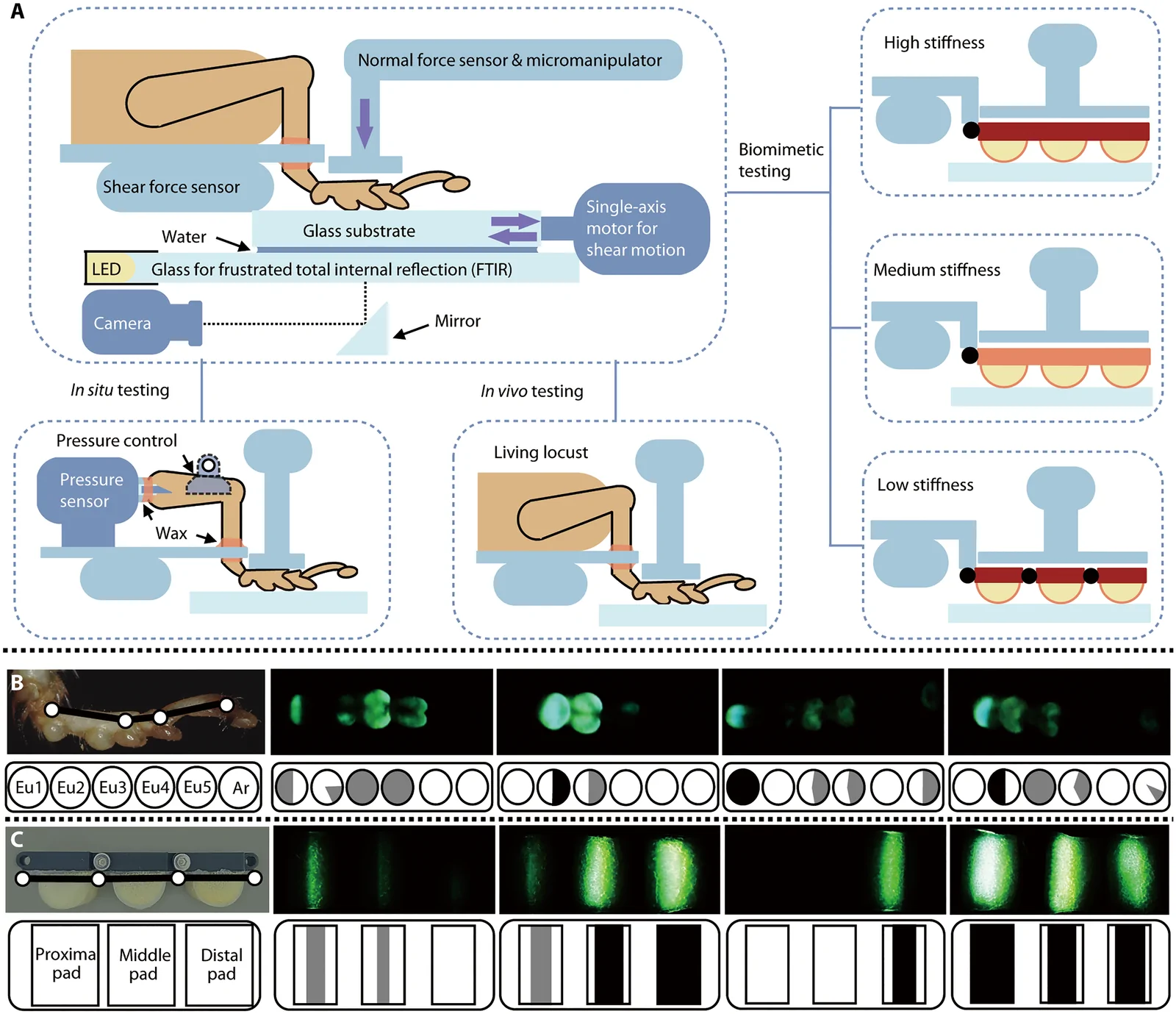
Schematic of the experimental design and the comparison of contact behaviors. (**A**) Components of the experimental platform: shear force testing, normal force testing, real contact area testing, and inner pressure testing. In situ testing: biomechanical testing for the in situ locust hindleg. In vivo testing: biomechanical testing for the in vivo locust hindleg. Biomimetic testing: mechanical testing of tarsal-inspired structures with high stiffness, medium stiffness, and low stiffness. (**B**) Actual contact images of the tarsus under several distinct states and their corresponding schematic contact diagrams; FTIR images and qualitative evaluation of dynamic frictional behavior in tarsal segments via contact interface monitoring: grayscale intensity correlates with contact pressure and geometric parameters encode contact area and location. (**C**) Actual contact images of the tarsal-inspired structures under several distinct states and their corresponding schematic contact diagrams; FTIR images and qualitative evaluation of dynamic frictional behavior. Ar: arolium, Eu: euplantular pad.

All biomechanical measurements (normal force, shear force, and hemolymph pressure) were acquired using a BIOPAC system (MP100A-CE, BIOPAC Systems Ltd., Santa Barbara, California, USA) with force sensors (FORT-100, 100 g capacity; FORT-1000, 1000 g capacity; FORT-5000, 5000 g capacity, Santa Barbara, California, USA) and blood pressure transducer (BLPR, World Precision Instrument, Sarasota, Florida, USA), following manufacturer calibration protocols. Force-time and pressure-time profiles were acquired using software (AcqKnowledge 3.7.0, BIOPAC Systems Ltd., Santa Barbara, California, USA) to quantify dynamic interaction during interfacial motion between the specimen and substrate. The normal force sensor was mounted on a micromanipulator (DC3001R, World Precision Instrument, Sarasota, Florida, USA) and equipped with a custom 3D-printed component to apply the force on the dorsal side of the sample. The friction forces between the dorsal cuticle of the locust tarsus and the 3D-printed component (fig. S8A), as well as between the baseplate of the tarsal-inspired structure and the 3D-printed component (fig. S8B), under different preloads are relatively small, indicating that the influence of these frictions on the experimental results can be ignored. The shear motion of the glass substrate was controlled by a single-axis motor (M-229.26S, Physik Instrumente SE & Co. KG, Karlsruhe, Germany).

The custom FTIR for contact area measurement comprised an LED light sheet, smooth glass, and water. The selected glass substrate in contact with the specimen eliminated confounding factors from tibial spine engagement due to the negligible friction of spines on the smooth glass. The deionized water between the glass substrate and the glass for FTIR was utilized as both optical couplant and interface lubricant. The real interfacial contact areas were determined through digital image acquisition (50 fps, D5300, Nikon, Tokyo, Japan) with a macro lens (Sigma EX 105 mm/2.8 DG Macro, SIGMA GmbH, Rödermark, Germany) and the contact area-time curves were quantified using an open-source image processing program (ImageJ v1.54f, the National Institutes of Health and the Laboratory for Optical and Computational Instrumentation in University of Wisconsin, USA) with threshold-based segmentation (IsoData method).

As shown in fig. S7, the bottom smooth glass designed for FTIR was securely mounted on a stationary frame. The reciprocating glass substrate was mounted on the single-axis motor (M-229.26S), with its surface subjected to a standardized cleaning protocol using anhydrous ethanol prior to each test to ensure consistent interfacial conditions. The test specimen was affixed to the shear force sensor’s clamping assembly, with its distal frictional components carefully adjusted to establish and maintain precise parallel contact with the substrate surface. The locust hindleg was mounted with the tibia fixed perpendicular to the substrate (beeswax immobilization), isolating tarsal movement driven by the femoral-tibial joint rotation. When integrating precision-calibrated hydraulic pressure sensors into the in situ locust hindleg setup, rigorous bubble removal and hermetic sealing are required to ensure measurement fidelity and to prevent pressure fluctuations during biomechanical characterization. The normal force applied to the samples was controlled by the micromanipulator (DC3001R) and measured by the normal force sensor. During the precision platform installation protocol, critical calibration procedures were implemented to: (i) achieve coaxial alignment between the shear force measurement axis and shear actuator’s displacement vector (ii) to establish orthogonal orientation of the normal force measurement axis relative to the substrate surface while maintaining strict collinearity with the geometric axis of frictional components, and (iii) and to verify optical alignment of the 45° mirror-coupled camera assembly in FTIR ensuring parallelism between its imaging axis and the substrate’s displacement trajectory for synchronized dynamic monitoring. The key behaviors identified in the controlled experiments are consistent with observations from the biological system. This cross-consistency indicates that the essential mechanics are captured by the present approach. Therefore, while simplified, the experimental design provides meaningful and transferable insight into the principles underlying locust jumping.

In this study, the distal ends of the locust tarsus and the tarsal-inspired structures always faced right (Fig. 9, B and C). Based on the jumping process of desert locusts, the three-phase testing sequence was: applying a normal force on the specimens and holding it (Step 1), pulling the substrate to the right (Step 2), and pushing the substrate to the left (Step 3). Notably, maintaining contact in the experimental setup is necessary to ensure repeatable and quantifiable interfacial conditions, allowing continuous measurement of force transmission and contact evolution. Without controlled contact, rapid and uncontrolled detachment events would obscure the underlying mechanical relationships between structure, pressure, and friction, making quantitative analysis difficult. As reported in previous studies, frictional performance is typically rate-dependent (*51*–*53*). Therefore, in this study, the pulling velocity for Step 2 and Step 3 was standardized at 2.0 mm/s in the biomechanical experiments, and at 2.5 mm/s in the experiments of tarsal-inspired structures. Force vectors (purple arrows, Fig. 9A) were monitored via normal and shear force sensors, while FTIR-based contact area quantification (movies S2 to S4) resolved pad-substrate interactions. Given that the hinged structures of the locust hindleg tarsus and the tarsal-inspired structures exhibit significantly greater mobility within a specific 2D plane compared to other directions, this study employs simplified 2D models to characterize their kinematics (Figs. 3, C to F, 4, C to F, and 7, C to G). Furthermore, the dynamic frictional behavior in each segment was qualitatively evaluated by contact interface monitoring, with gray-scale intensity correlated to contact pressure and geometric parameters encoding contact area and position. In this study, the in situ experiments were conducted on seven individuals. One complete dataset obtained under stable experimental conditions throughout the full testing sequence was used for the quantitative analysis presented in the main text. Selected additional datasets from independent individuals are provided in fig. S9 to illustrate results observed in other preparations. For the in vivo tests, a single healthy locust was selected and examined under two different preloads. Three experimental replicates were conducted for each preload condition, and the results of the remaining two replicates are displayed in fig. S10.
