# Supplementary material for: Hemolymph pressure-mediated friction in the tarsus of the desert locust (Schistocerca gregaria)
Source: Sci Adv. 2026 Oct 23;12(43):eaeg9427. doi: 10.1126/sciadv.aeg9427 (PMC13693668; doi:10.1126/sciadv.aeg9427)
Supplement: Supplementary file 1 [file sciadv.aeg9427_sm.pdf]

Science Advances
AAAS

# Supplementary Materials for

## Hemolymph pressure-mediated friction in the tarsus of the desert locust (*Schistocerca gregaria*)

Jiahui Zhao *et al.*

Corresponding author: Zhendong Dai, zddai@nuaa.edu.cn; Chuchu Li, cli@zoologie.uni-kiel.de

*Sci. Adv.* **12**, eaeg9427 (2026)
DOI: 10.1126/sciadv.aeg9427

**The PDF file includes:**

Figs. S1 to S10
Legends for movies S1 to S4

**Other Supplementary Material for this manuscript includes the following:**

Movies S1 to S4

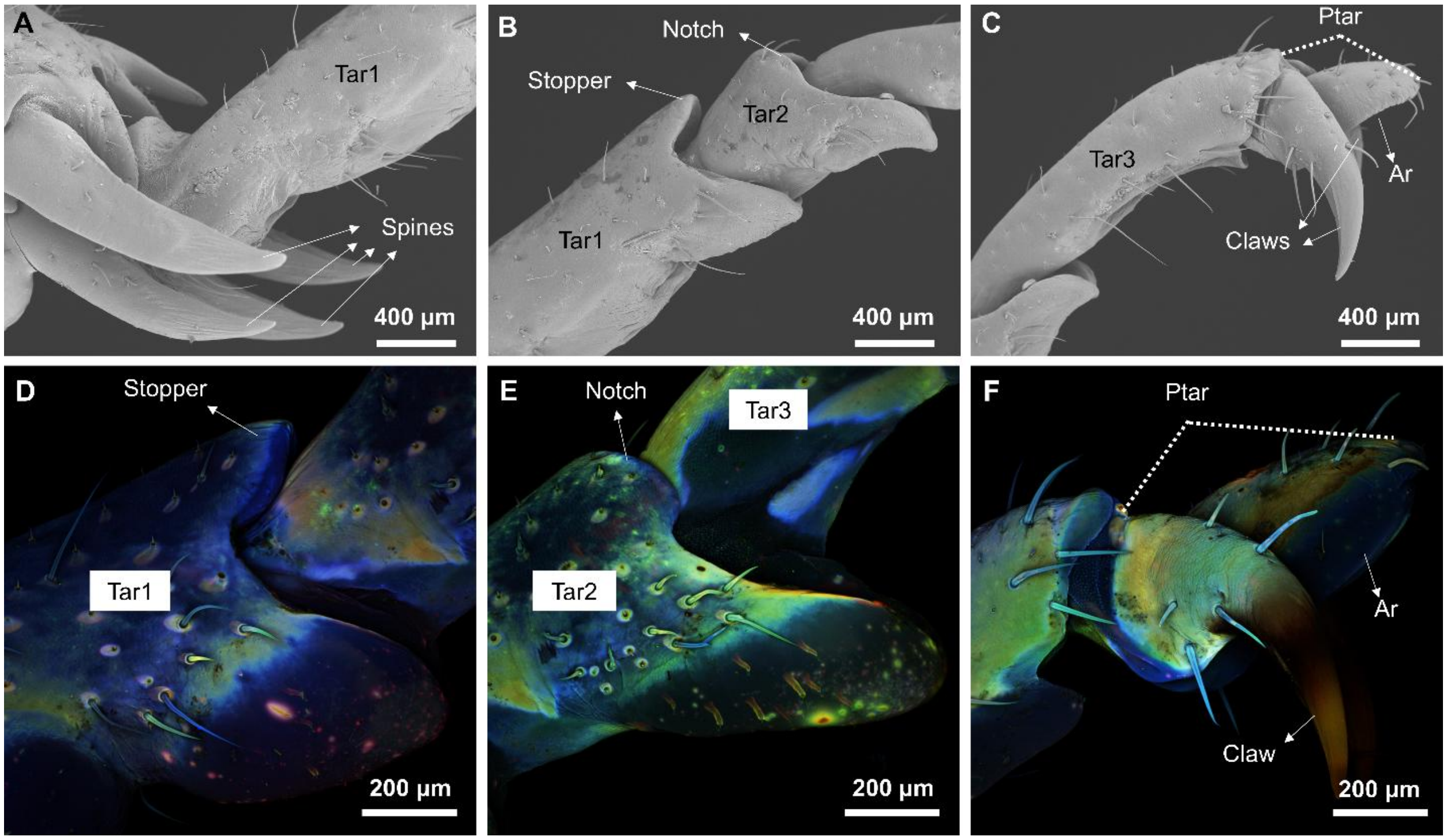


**Fig. S1.**

**Morphology of the tarsal joints in the locust hindleg.** (**A–C**) SEM of lateral articular morphology shows the cuticular stopper between Tar1 and Tar2. (**D–F**) CLSM of cuticular architecture shows a reduced sclerotization of the stopper.

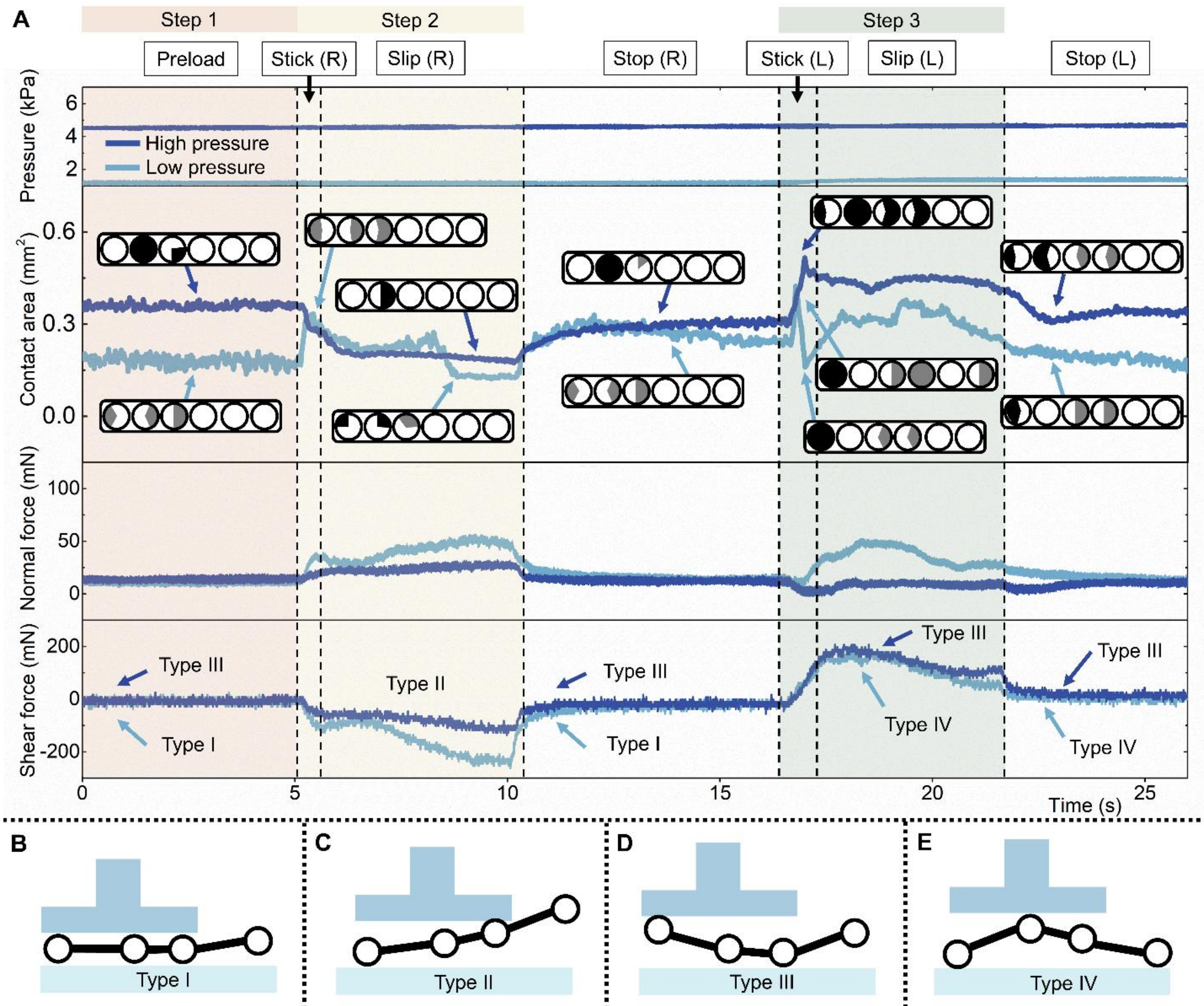


**Fig. S2.**

***In situ* locust hindleg tarsus.** Dynamic variations in contact area, normal force, and shear force parameters were systematically investigated through hemolymph pressure modulation during complete experimental cycles (10 mN preload).

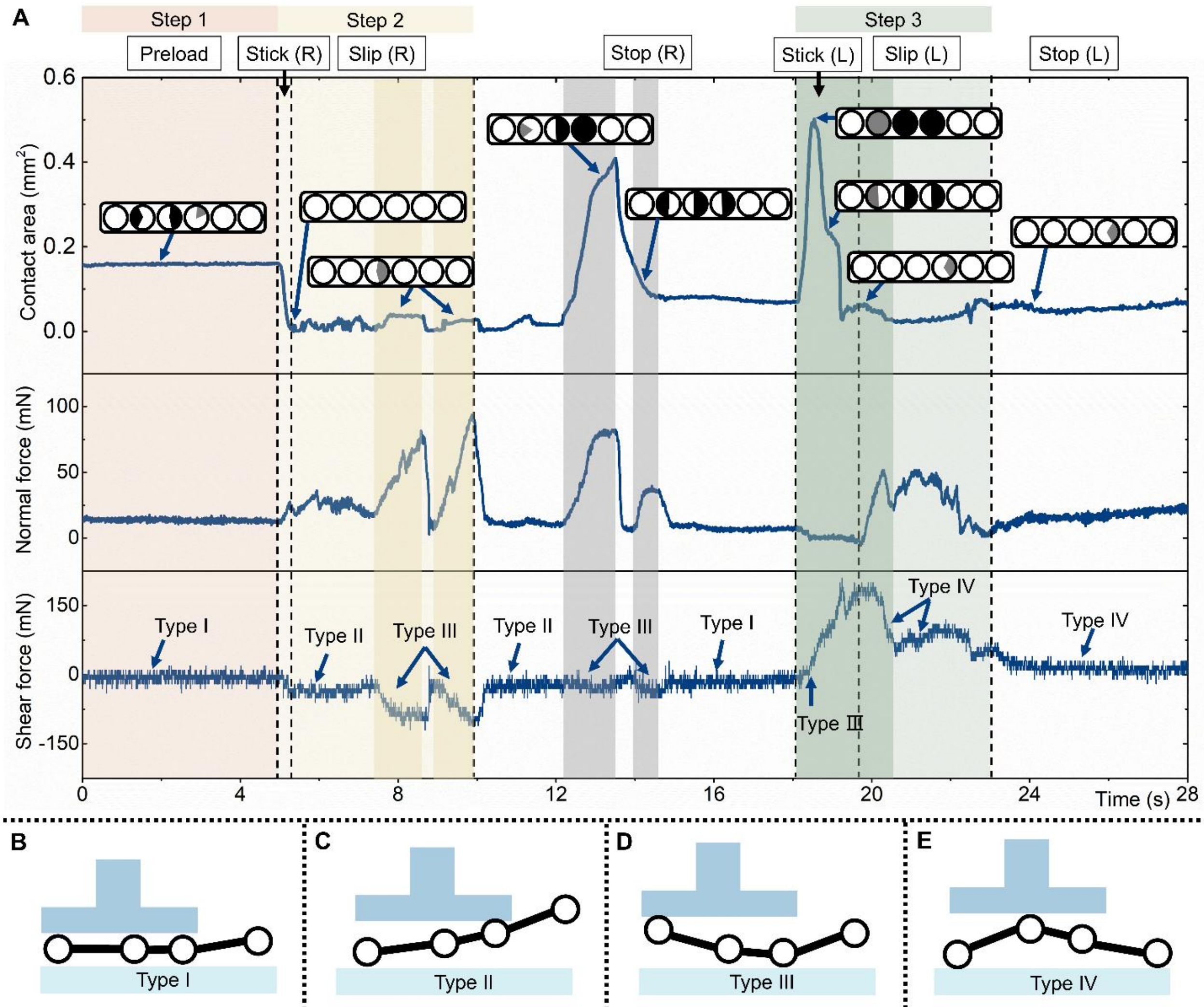


**Fig. S3.**

***In vivo* locust hindleg tarsus.** The impact of dynamic modulation of autologous hemolymph pressure on variations in tarsal contact area, normal forces, and shear forces throughout a comprehensive experimental cycle (10 mN preload).

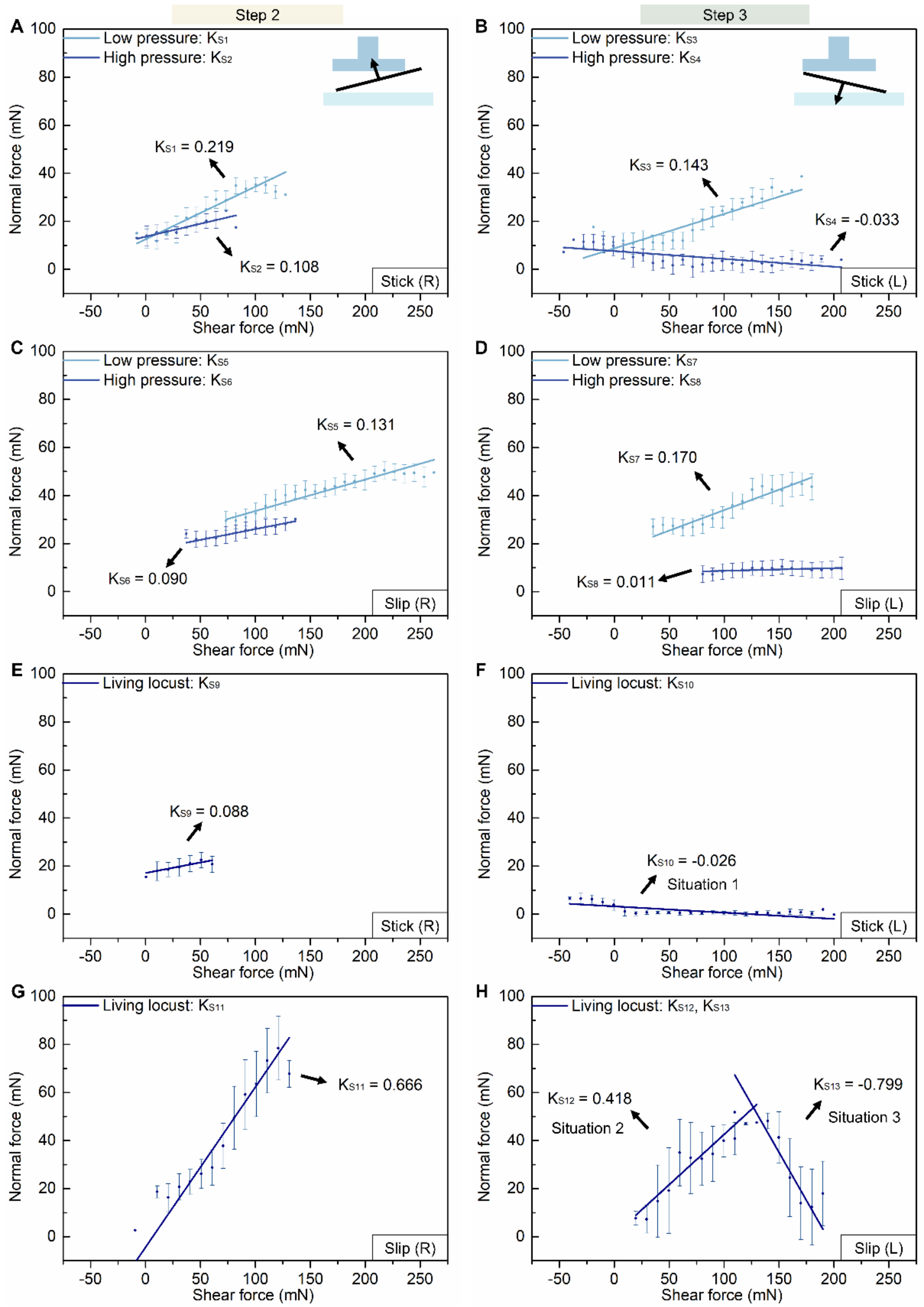


**Fig. S4.**

**Normal-shear force relationships of *in situ* and *in vivo* tarsi under 10 mN preload.** (**A–D**) The relationships between the normal forces and the shear forces of the *in situ* locust tarsus during Step 2 and Step 3. (**E–H**) The relationships between the normal forces and the shear forces of the *in vivo* locust tarsus during Step 2 and Step 3.

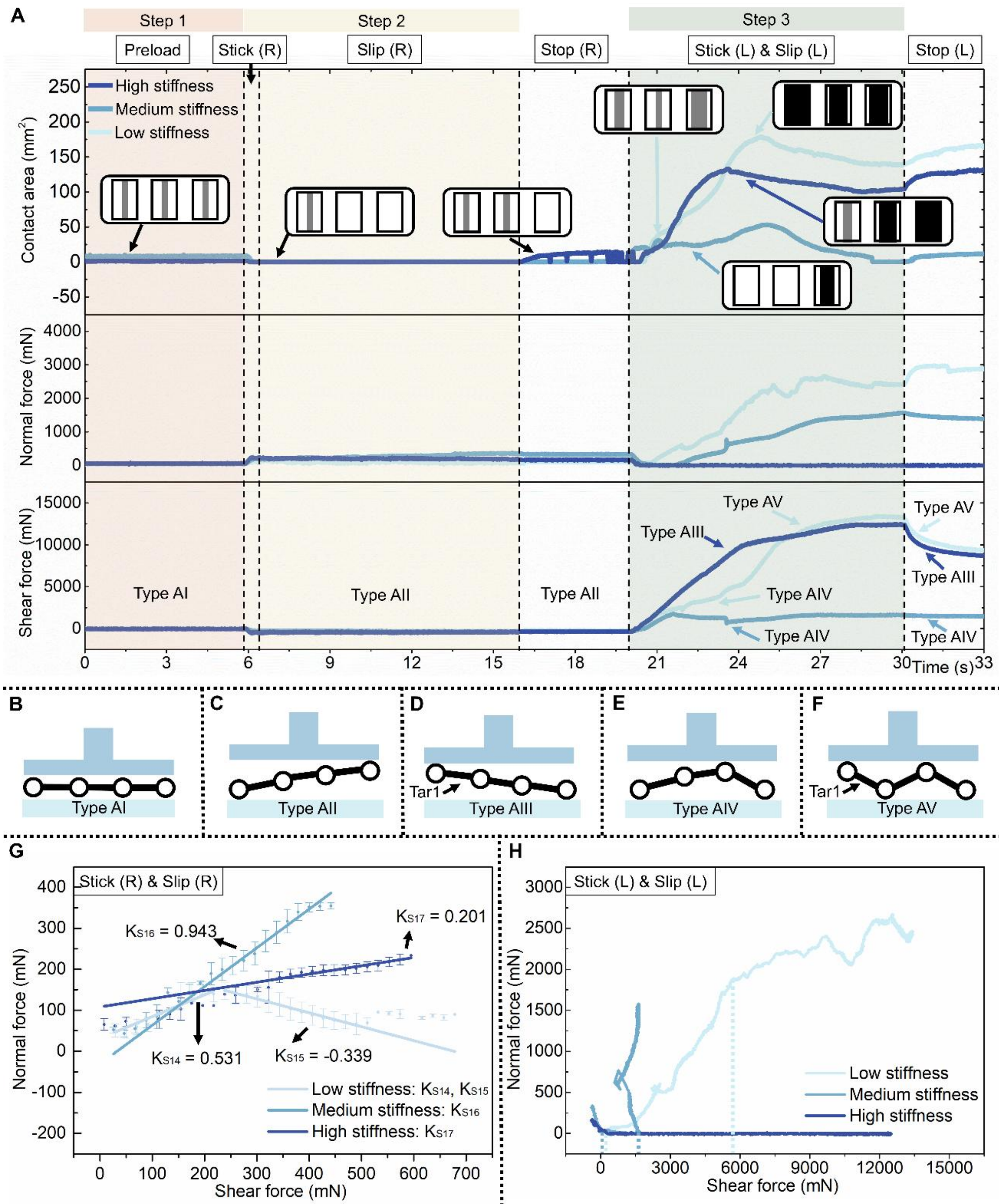


**Fig. S5.**

**Tarsal-inspired structures.** (**A**) Dynamic variations of contact area, normal force, and shear force to baseplate stiffness modulation in tarsal-inspired structures throughout a complete experimental cycle (50 mN preload). (**B–F**) Quasi-static 2D modeling of tarsal-inspired structures with five distinct morphologies as Type AI–AV. (**G, H**) The effects of baseplate stiffness variations on normal-shear force relationships during Step 2 and Step 3 (50 mN preload).

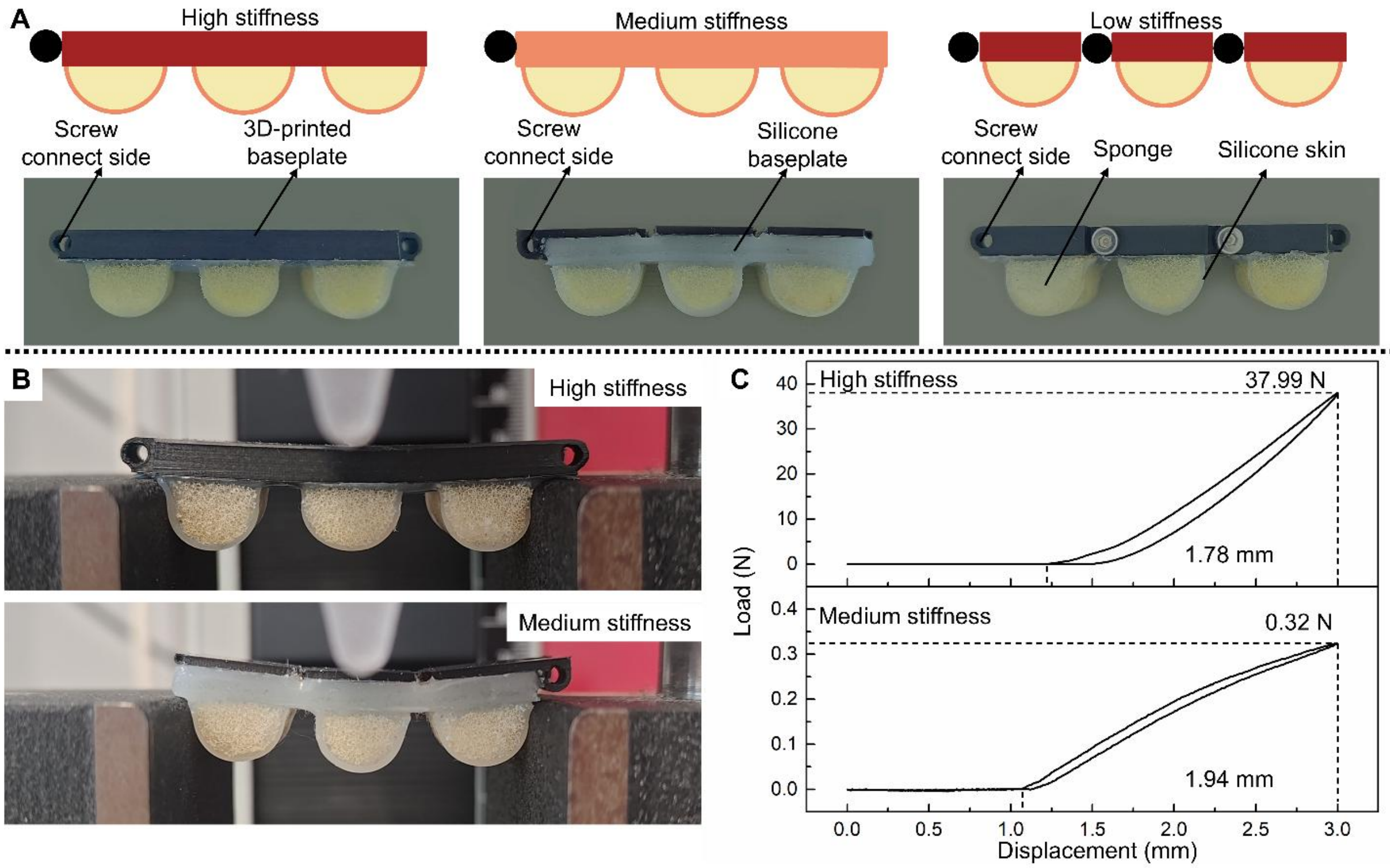


**Fig. S6.**

**Tarsal-inspired structures.** (**A**) Schematic diagram of three tarsal-inspired structures with corresponding physical illustrations. (**B, C**) The three-point bending test results of high-stiffness and medium-stiffness structures.

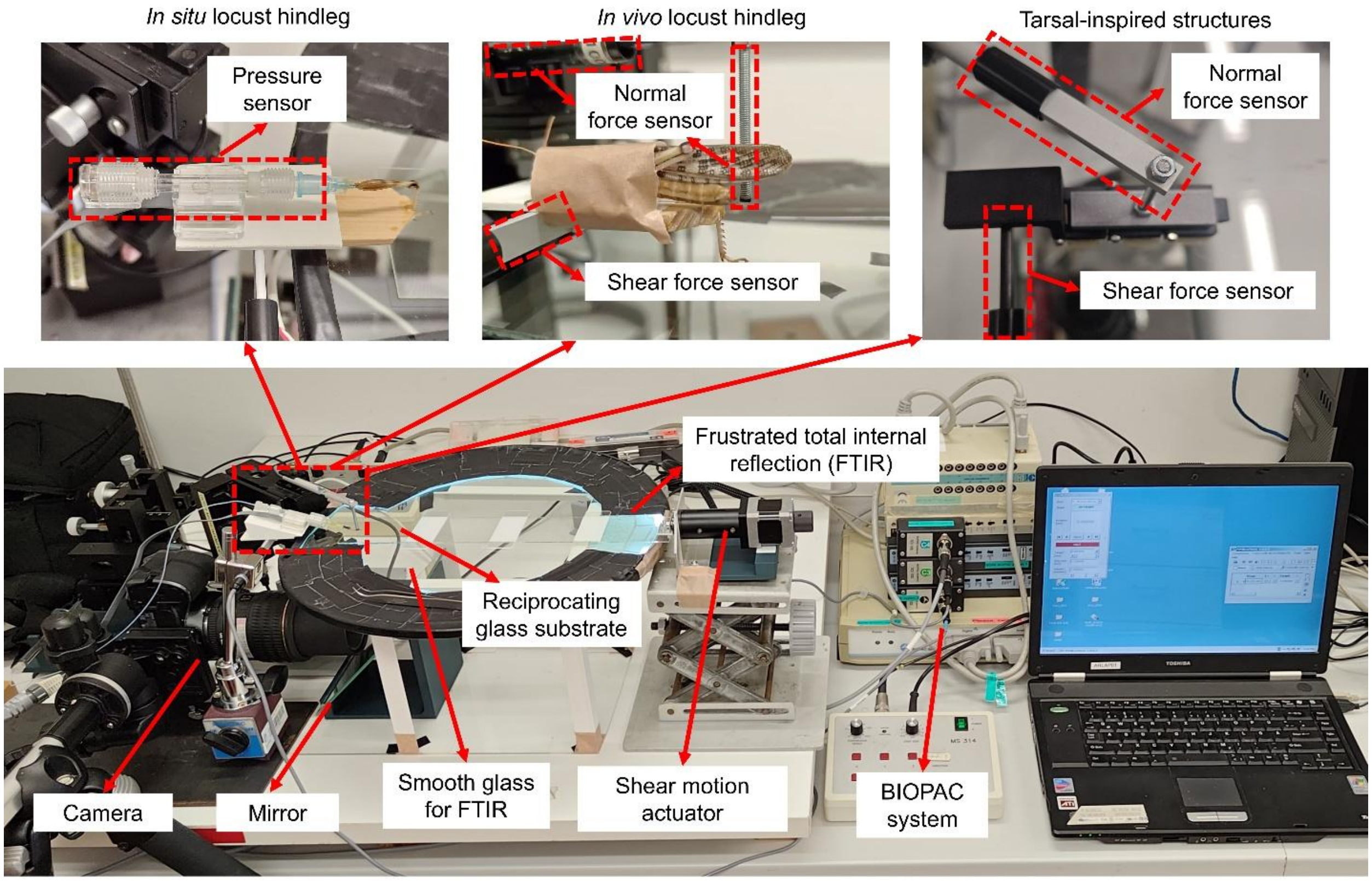


**Fig. S7.**

Physical images of the experimental platform.

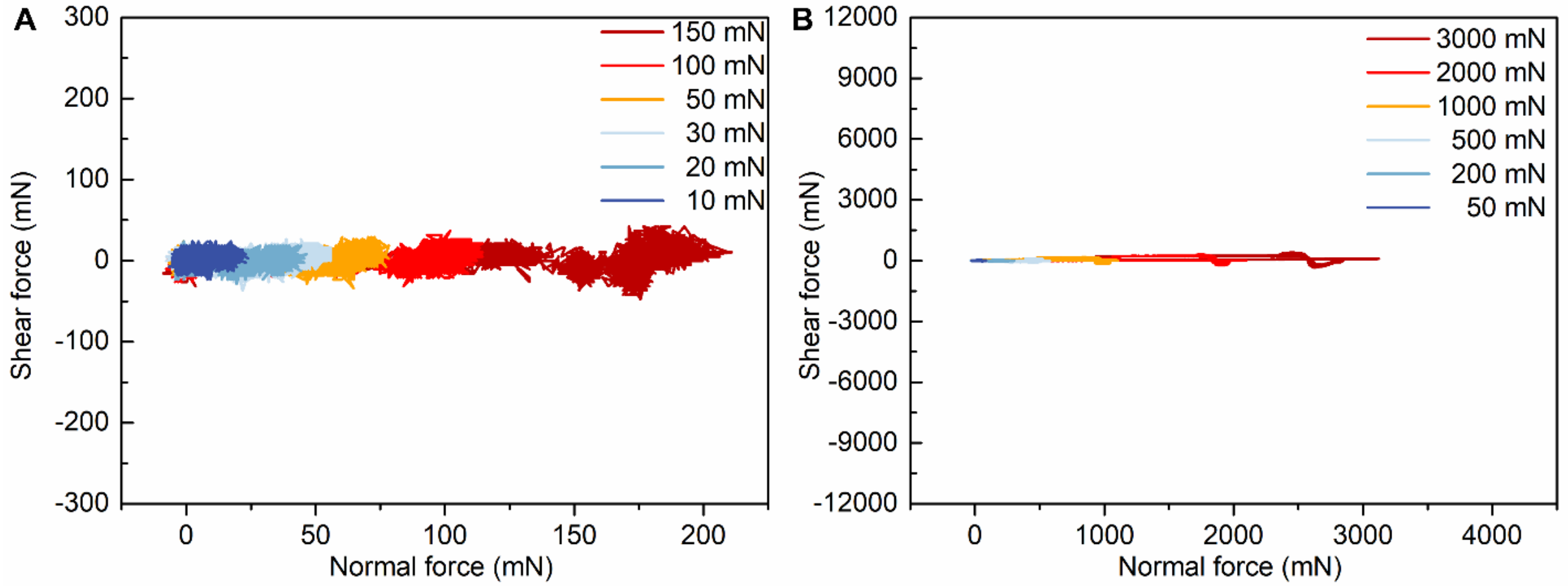


**Fig. S8.**

(**A**) Friction between the dorsal cuticle of the locust tarsus and 3D-printed custom component under different preloads. (**B**) Friction between the baseplate of the tarsal-inspired structure and the 3D-printed custom component under different preloads.

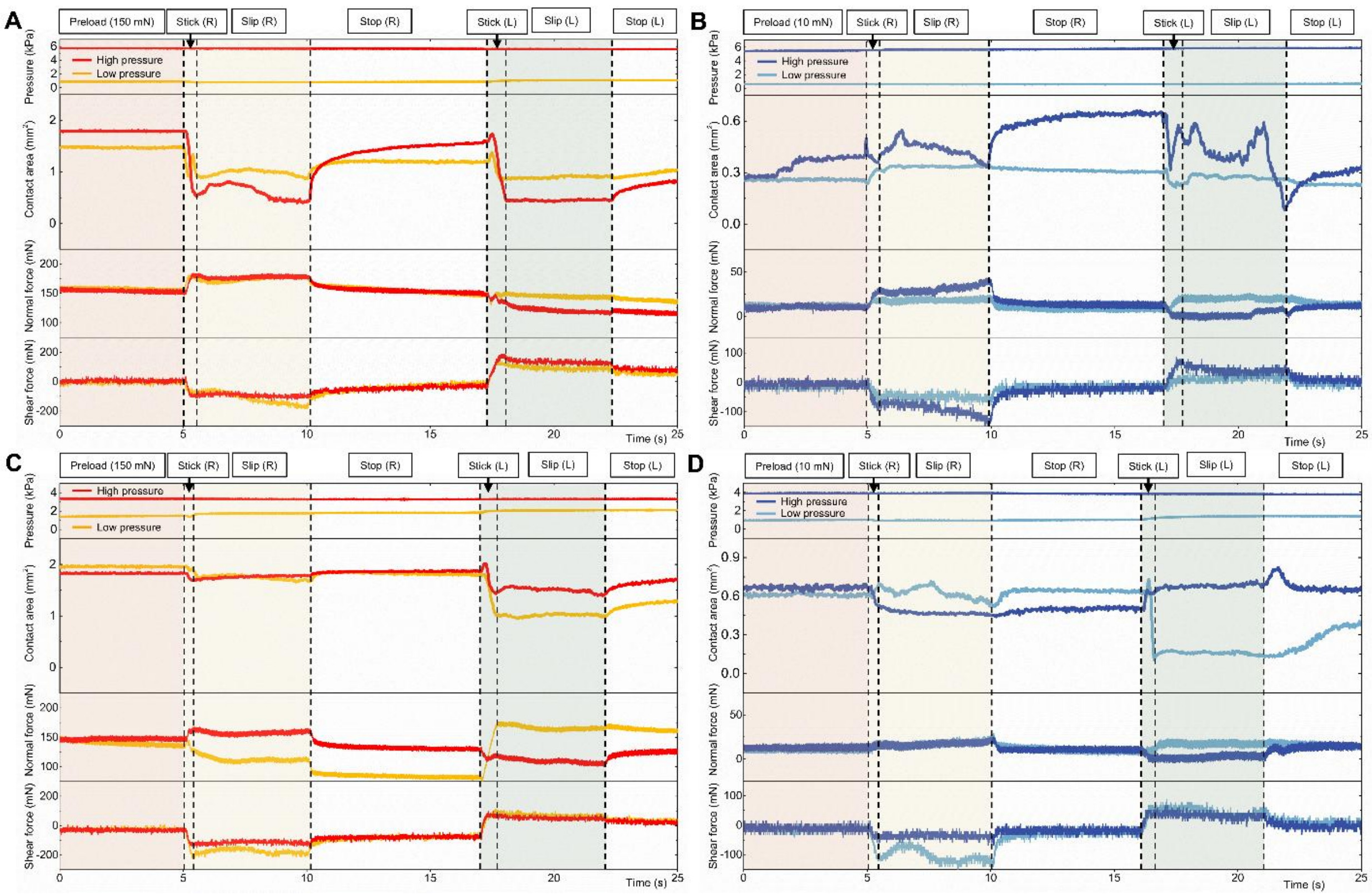


**Fig. S9. Additional outcomes from independent *in situ* preparations.**

Experimental results obtained from two additional locust individuals. (**A, B**) Results from one preparation in which slipping associated with the secretion layer occurred during the stationary phase of Step 3, affecting the normal-force and contact-area responses. (**C, D**) Results from another preparation in which hemolymph leakage occurred under the high normal force, high pressure condition, preventing stable maintenance of the imposed pressure. These datasets are shown to illustrate outcomes from additional preparations and were not used for the quantitative analysis presented in the main text.

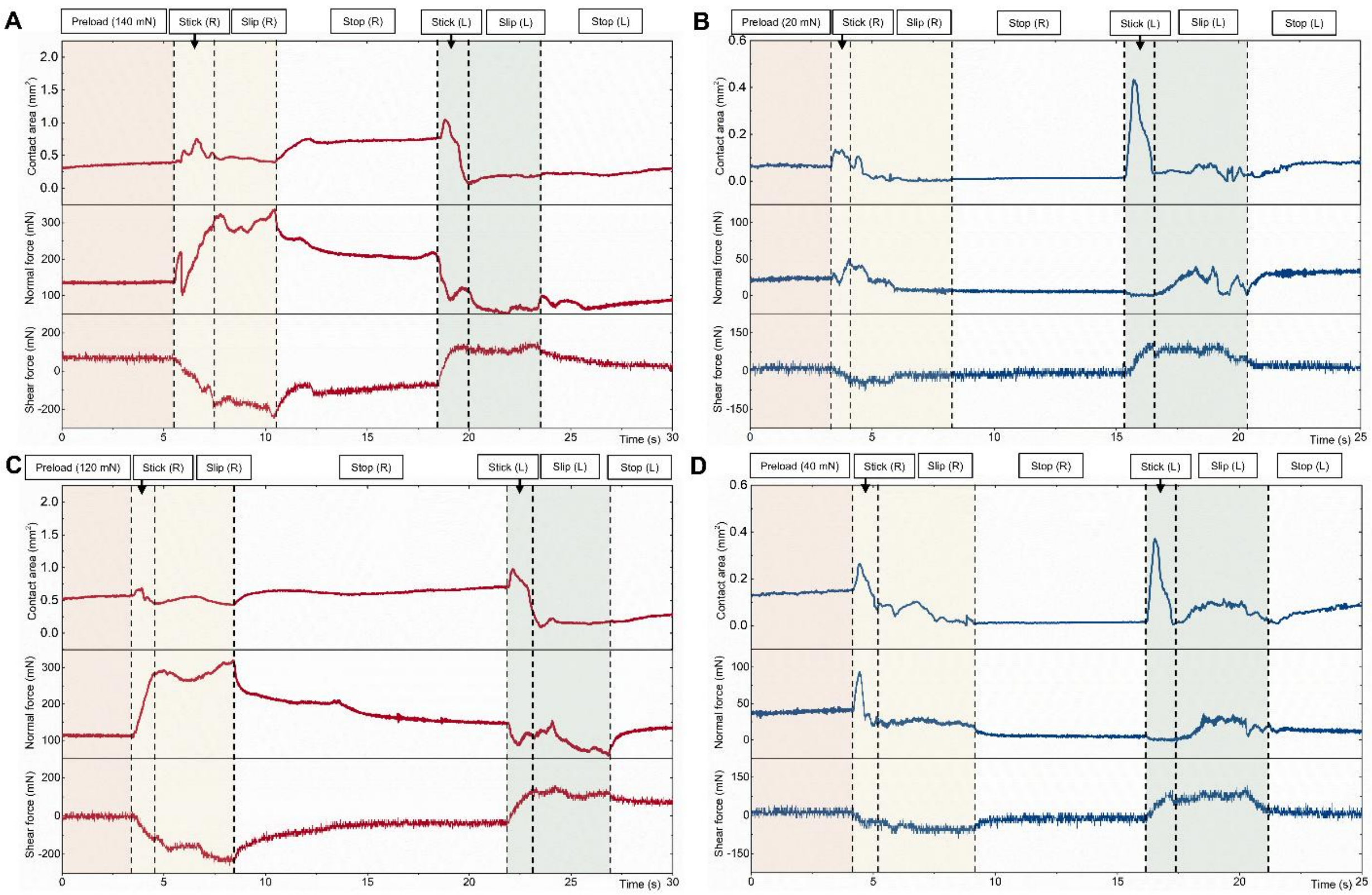


**Fig. S10.**

Experimental results of the second (**A**, **B**) and third (**C**, **D**) replicates from repeated *in vivo* tests on a healthy locust.

**Movie S1.**
Different motions of the desert locust hindleg tarsus.

**Movie S2.**
Dynamic variations in contact area of *in situ* locust hindleg tarsi.

**Movie S3.**
Dynamic variations in contact area of *in vivo* locust hindleg tarsi.

**Movie S4.**
Dynamic variations in contact area of tarsal-inspired structures.
